# Systematic review of research design and reporting of imaging studies applying convolutional neural networks for radiological cancer diagnosis

**DOI:** 10.1007/s00330-021-07881-2

**Published:** 2021-04-16

**Authors:** Robert J. O’Shea, Amy Rose Sharkey, Gary J. R. Cook, Vicky Goh

**Affiliations:** 1 Cancer Imaging, School of Biomedical Engineering and Imaging Sciences, King’s College London, 5th floor, Becket House, 1 Lambeth Palace Road, London, SE1 7EU UK; 2 Department of Radiology, Guy’s & St Thomas’ NHS Foundation Trust, London, UK; 3 King’s College London & Guy’s and St. Thomas’ PET Centre, London, UK

**Keywords:** Artificial intelligence, Deep learning, Diagnosis, computer-assisted, Neoplasms, Research design

## Abstract

**Objectives:**

To perform a systematic review of design and reporting of imaging studies applying convolutional neural network models for radiological cancer diagnosis.

**Methods:**

A comprehensive search of PUBMED, EMBASE, MEDLINE and SCOPUS was performed for published studies applying convolutional neural network models to radiological cancer diagnosis from January 1, 2016, to August 1, 2020. Two independent reviewers measured compliance with the Checklist for Artificial Intelligence in Medical Imaging (CLAIM). Compliance was defined as the proportion of applicable CLAIM items satisfied.

**Results:**

One hundred eighty-six of 655 screened studies were included. Many studies did not meet the criteria for current design and reporting guidelines. Twenty-seven percent of studies documented eligibility criteria for their data (50/186, 95% CI 21–34%), 31% reported demographics for their study population (58/186, 95% CI 25–39%) and 49% of studies assessed model performance on test data partitions (91/186, 95% CI 42–57%). Median CLAIM compliance was 0.40 (IQR 0.33–0.49). Compliance correlated positively with publication year (*ρ* = 0.15, *p* = .04) and journal H-index (*ρ* = 0.27, *p* < .001). Clinical journals demonstrated higher mean compliance than technical journals (0.44 vs. 0.37, *p* < .001).

**Conclusions:**

Our findings highlight opportunities for improved design and reporting of convolutional neural network research for radiological cancer diagnosis.

**Key Points:**

• *Imaging studies applying convolutional neural networks (CNNs) for cancer diagnosis frequently omit key clinical information including eligibility criteria and population demographics.*

• *Fewer than half of imaging studies assessed model performance on explicitly unobserved test data partitions.*

• *Design and reporting standards have improved in CNN research for radiological cancer diagnosis, though many opportunities remain for further progress.*

**Supplementary Information:**

The online version contains supplementary material available at 10.1007/s00330-021-07881-2.

## Introduction

Recent years have seen an increase in the volume of artificial intelligence (AI) research in the field of cancer imaging, prompting calls for appropriately rigorous design and appraisal standards [1–6]. Evaluation of AI research requires a skillset which is distinct from those of classical medical statistics and epidemiology. The problems of high dimensionality, overfitting and model generalisation are central challenges in AI modelling [7–10]. These phenomena potentially compromise the generalisation of AI models to the reality of clinical practice [11]. However, the reliability of these models may be estimated and maximised through rigorous experimental design and reporting [1, 12].

EQUATOR was founded to improve the quality of scientific research through standardisation of reporting guidelines [13, 14]. Established EQUATOR guidelines such as STARD [15], STROBE [16] and CONSORT [17] were not designed specifically to address the challenges of AI research. AI-focused guidelines have recently been developed including CLAIM [18], SPIRIT-AI [19], MI-CLAIM [20] and, prospectively, STARD-AI [21]. These are welcome measures as AI remains at an early phase of clinical implementation for diagnostic tasks. Although each set of reporting standards addresses a specific task, a high degree of overlap exists between these guidelines, reflecting the fundamental importance of many of the criteria.

CLAIM aims to promote clear, transparent and reproducible scientific communication about the application of AI to medical imaging and provides a framework to assure high-quality scientific reporting. Current conformity to these standards has not been formally quantified to date. Consequently, a need exists for a contemporary evaluation of design and reporting standards in the domain of cancer imaging AI research.

Following ImageNet 2012 [22], convolutional neural network (CNN) models have been adapted to various biomedical tasks. The approach is now the industry standard in AI applications for diagnostic radiology [23, 24]. In this study, we aim to quantify explicit satisfaction of the CLAIM criteria in recent studies applying CNNs to cancer imaging. We examine the adequacy of data and ground truth collection, model evaluation, result reporting, model interpretation, benchmarking and transparency in the field. We identify key areas for improvement in the design and reporting of CNN research in the field of diagnostic cancer imaging.

## Materials and methods

### Inclusion criteria

1. The article evaluates a CNN model for radiological cancer diagnosis in humans.
2. The model receives a radiological image as its sole input.
3. The article was published in a peer-reviewed journal between January 1, 2016, and August 1, 2020.
4. The article is published in the English language.

### Exclusion criteria

1. The model addresses a non-diagnostic task such as pre-processing, segmentation or genotyping.
2. The model receives non-radiological images such as histopathology, dermoscopy, endoscopy or retinoscopy.
3. The article presents experiments on animal or synthetic data.
4. The article primarily addresses economic aspects of model implementation.
5. The article is published in a low-impact journal.
6. The article is unavailable in full-text format.

### Search

PubMed, EMBASE, MEDLINE and SCOPUS databases were searched systematically for original articles from January 1, 2016, to August 14, 2020, for articles meeting our inclusion and exclusion criteria. Search queries for each database are included in the supplementary material. The search was performed on August 14, 2020. No other sources were used to identify articles. Screening and decisions regarding inclusion based on the full text were performed independently by 2 reviewers (R.O.S., A.S., clinical fellows with 3 years and 1 year of experience of AI research, respectively) and disagreements resolved by consensus. A senior reviewer (V.G.) was available to provide a final decision on unresolved disagreements. Duplicated articles were removed. Articles were not screened with the QUADAS tool [25], as it shares several items with the CLAIM guideline. Exclusion of QUADAS-incompliant articles would have biased subsequent estimations of CLAIM compliance.

### Data extraction

Data items were defined to measure compliance with CLAIM proposal and previously published proposals [1, 18]. Complex items with multiple conditions were subdivided as appropriate. Data items are listed in Table 1. First author, journal, publication year, modality and body system were also extracted. Studies which served to validate existing models were exempt from all items pertaining to model development. Studies not employing model ensembling were exempt from item 27.

**Table 1:** List of the data items evaluated. Items are derived from the CLAIM guidance. CLAIM items with multiple conditions are divided into sub-items, denoted as alphabetical suffixes. Compliant values are all values considered satisfactory for that item. Exemptions indicate types of study which are not required to satisfy an item

Item	Criterion	Values	Compliant values	Exemptions
1	Title or abstract specified application of convolutional neural network model	0. Not specified 1. Specified	1	None
2	Abstract included summary of study design, methods, results and conclusions	0. Not included 1. Included	1	None
3	Introduction provided scientific and clinical background with role for model	0. Not provided 1. Provided	1	None
4a	Study objectives	0. Not provided 1. Provided	1	None
4b	Study hypotheses	0. Not documented 1. Documented	1	None
5	Indicated prospective or retrospective study timeframe	0. Not documented R. Retrospective P. Prospective RP. Both retrospective and prospective	R, P, RP	None
6	Study goal	0. Not documented 1. Documented	1	None
7a	Data source	0. Not documented L. Local data collection P. Public data LP. Both local and public data	L, P, LP	None
7b	Data collection institutions	0. Not documented SC. Single-centre data MC. Multi-centre data	SC, MC	None
7c	Imaging equipment vendors	0. Not documented SV. Single vendor MV. Multiple vendors	SV, MV	None
7d	Image acquisition parameters	0. Not documented 1. Documented	1	None
7e	Institutional review board approval	0. Not documented 1. Documented	1	None
7f	Participant consent	0. Not documented 1. Documented	1	None
8	Eligibility criteria	0. Not documented 1. Documented	1	None
9	Image pre-processing	0. Not documented P. Pre-processing documented PM. Reproducible pre-processing method documented NP. Documented that pre-processing not employed	PM, NP	None
10	Data subsetting	0. Not documented C. Image cropping documented CM. Reproducible image cropping method documented NC. Documented that cropping not employed	CM, NC	None
11	Model predictors and outcomes	0. Not defined 1. Not defined	1	None
12	Data de-identification	0. Not documented A. Anonymisation documented AM. Reproducible anonymisation method documented	AM	None
13	Missing data handling strategy	0. Not documented E. Missing data excluded from analysis I. Missing data included in analysis	E, I	None
14	Reference standard definition	0. Not defined 1. Defined either explicitly or by reference to a Common Data Element such as the American College of Radiology Image Reporting and Data Systems.	1	None
15a	Reference standard rationale	0. Not documented 1. Documented	1	None
15b	Definitive ground truth	0. No definitive ground truth P. Histopathology DI. Definitive imaging modality FU. Case follow-up PFU. Histopathology and case follow-up PDI. Histopathology and definitive imaging modality	P, DI, FU, PFU, PDI	None
16a	Manual image annotation	0. Not documented UR. Radiologist with unspecified expertise SR. Radiologist with relevant subspecialist expertise OC. Other clinician	SR	None
16b	Histopathology annotation	0. Not documented SP. Pathologist with relevant subspecialist expertise	SP	Histopathology not employed
17	Image annotation tools and software	0. Not documented 1. Documented	1	None
18	Annotator variability	0. Not documented V. Variability statistics documented M. Aggregation method documented VM. Variability statistics and aggregation method documented	VM	None
19a	Sample size	0. Not documented 1. Documented number of images in dataset	1	None
19b	Provided power calculation	0. Not documented 1. Documented	1	None
19c	Distinct study participants	0. Not documented {N}. N = number of study participants	{N}	None
20	Data partitions and their proportions	0. Not documented 1. Documented	1	None
21	Partition disjunction	0. Not documented 1. Documented partition disjunction at patient level	1	Validation studies
22a	Provided reproducible model description	0. Not documented 1. Documented	1	Validation studies
22b	Provided source code	0. Not documented 1. Documented	1	Validation studies
23	Modelling software	0. Not documented S. Documented software SV. Documented software and version	SV	Validation studies
24	Parameter initialisation method	0. Not documented R. Random initialisation T. Transfer learning RT. Both random initialisation and transfer learning employed	R	Validation studies
25a	Provided reproducible data augmentation strategy or specified used of unaugmented data	0. Not documented A. Documented data augmentation AM. Reproducible data augmentation method NA. No data augmentation	AM, NA	Validation studies
25b	Loss function	0. Not documented 1. Documented	1	Validation studies
25c	Optimisation method	0. Not documented 1. Documented	1	Validation studies
25d	Learning rate settings	0. Not documented 1. Documented	1	Validation studies
25e	Stopping protocol for model training	0. Not documented 1. Documented	1	Validation studies
25f	Batch size	0. Not documented 1. Documented	1	Validation studies
26	Model selection	0. Not documented 1. Documented model selection criterion, specifying *k* if *k*-fold cross validation employed	1	Validation studies
27	If model ensembling applied, provided ensembling method	0. Not documented E. Ensembling documented EM. Documented reproducible ensembling method	EM	Ensembling not employed
28	Metrics	0. Not documented M. Defined performance metrics MR. Defined performance metrics and provided rationale	MR	None
29	Significance	0. Not documented S. Model significance documented SM. Model significance documented with reproducible methodology	SM	None
30	Robustness	0. Not documented 1. Documented model robustness to variation in experimental conditions such as sample size, noise and imaging equipment	1	None
31	Model interpretation	0. Not documented I. Interpreted model IM. Interpreted model with validated methodology	IM	None
32	Test data description	0. Not described I. Employed internal test data E. Described test data from different institution	I, E	None
33	Case-flow diagram	0. Not documented 1. Documented	1	None
34	Demographics and clinical characteristics	0. Documented D. Documented aggregate statistics DP. Documented statistics for each data partition	DP	None
35a	Test performance	0. Model performance assessed on data observed during training V. Model performance assessed on data observed during model selection T. Model performance assessed on data which was unobserved during training and model selection	T	None
35b	Human diagnostic performance benchmarking	0. No human performance benchmark UR. Benchmarked against radiologist with unspecified expertise SR. Benchmarked against radiologist with relevant subspecialist expertise OC. Benchmarked against other clinicians	SR	None
35c	Computational diagnostic performance benchmarking	0. No computational benchmark 1. Benchmarked against other computational methods	1	None
36	Diagnostic performance with measure of precision	0. Diagnostic performance reported without measure of precision 1. Diagnostic performance reported with confidence interval or standard error	1	None
37	Failure analysis	0. Not discussed 1. Discussed misclassified cases or model errors	1	None
38	Study limitations	0. Not discussed 1. Discussed	1	None
39	Clinical implications of study findings	0. Not discussed 1. Discussed	1	None
40	Study registration number	0. Not documented 1. Documented	1	None
41	Study protocol	0. Not documented 1. Provided access to the full study protocol	1	None
42	Funding	0. Not documented F. Funding source documented FR. Funding source and role documented NF. Stated no funding received	FR, NF	None

Articles were read and annotated by R.O.S. and A.S., and disagreements were resolved by consensus. Articles were read in random order, using a fixed sequence generated in R [26]. Journal H-index was extracted from the Scimago journal rankings database [27]. Journals were categorised as either “clinical” or “technical” according to the journal name—names containing any term related to computer science, artificial intelligence or machine learning were assigned the “technical” category. The remaining journals were assigned the “clinical” category.

### Data analysis

Statistical analysis was conducted using R version 3.5.3 [26] and RStudio version 1.1.463 [28]. For each item, the proportion of compliant studies was measured, excluding those with applicable exemptions. For items with ≥ 3 response categories, proportions were also measured for each category. Ninety-five percent confidence intervals (95% CI) were estimated around binary proportions using the method of Clopper and Pearson [29] and around multinomial proportions using the method of Sison and Glaz [30, 31]. Following adherence assessment recommendations [32], an overall CLAIM compliance score was defined per article by the proportion of applicable items satisfied. Items and subitems were weighted equally.

$$ \mathrm{CLAIM}\ \mathrm{compliance}=\frac{\mathrm{number}\ \mathrm{of}\ \mathrm{items}\ \mathrm{satisfied}}{\mathrm{number}\ \mathrm{of}\ \mathrm{items}\ \mathrm{applicable}} $$

Temporal change in CLAIM compliance was evaluated by two-sided test of Spearman rank correlation between CLAIM score and year of publication. Association between journal impact factor and compliance was evaluated with a two-sided test of Spearman rank correlation between journal H-index and CLAIM score. The difference in mean CLAIM compliance between clinical and technical journals was evaluated with a two-sided *t* test. All code and data required to support the findings of this research are available from the corresponding author upon request. As a methodological review assessing study reporting, this study was not eligible for registration with the PROSPERO database.

## Results

### Search

**Fig. 1:**
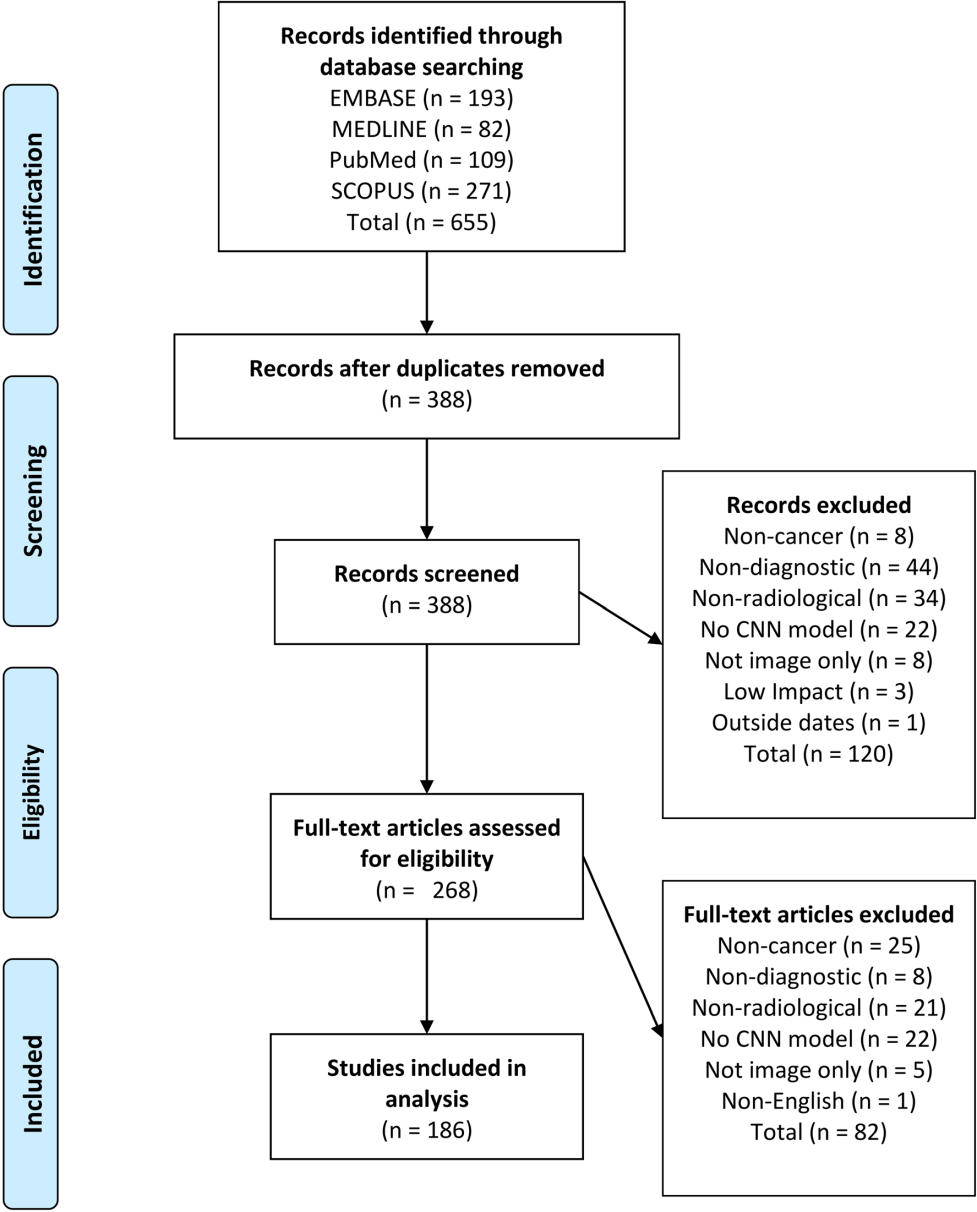
Flow diagram of literature search process

**Fig. 2:**
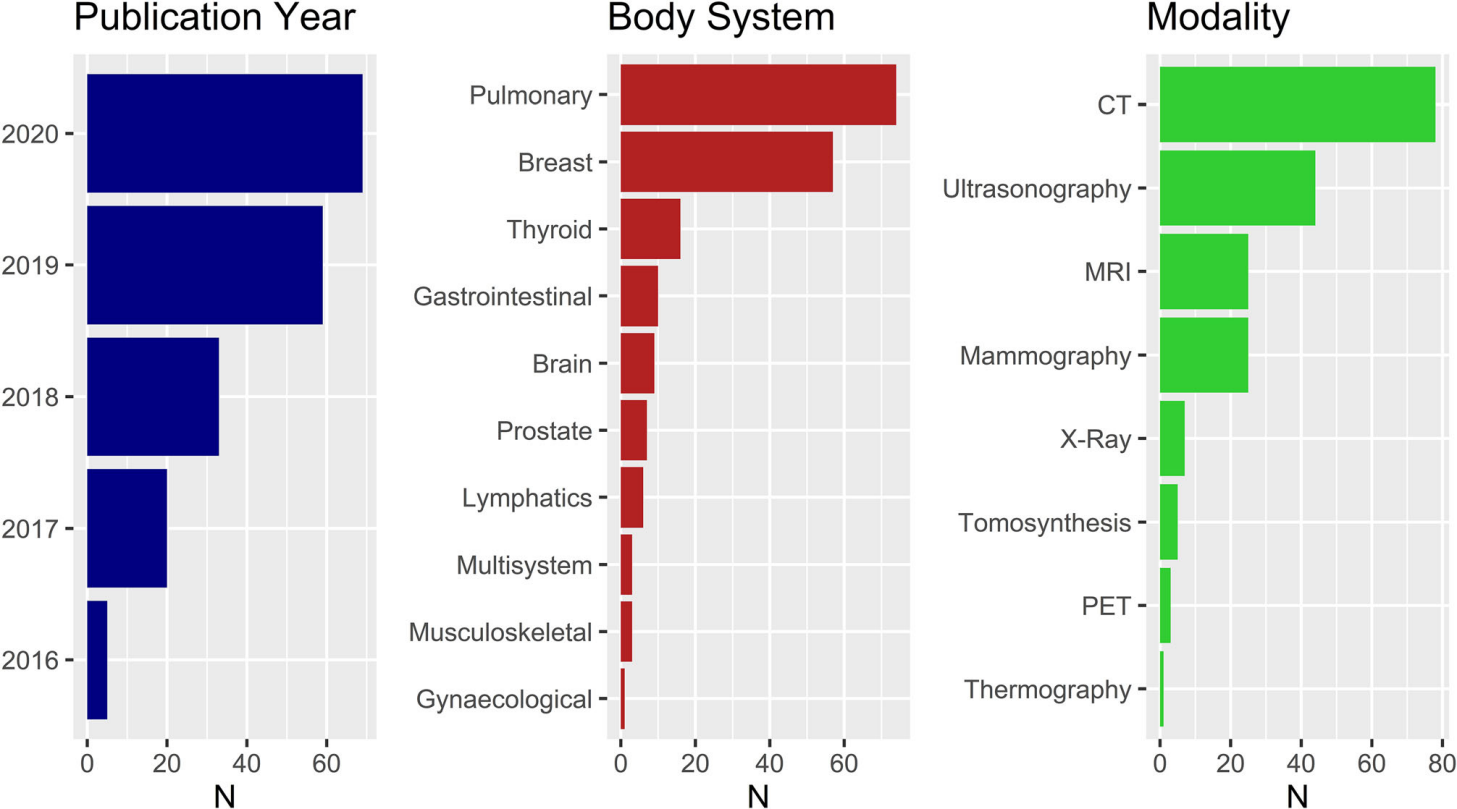
Distribution of included articles. Left: study publication year. Middle: body system imaged. Right: imaging modality employed

Six hundred fifty-five articles were identified in the primary database search, of which 267 were duplicates. One hundred twenty articles were excluded during title screen, and 82 articles were excluded during abstract screening. One hundred eighty-six articles were included in the final analysis. A flow diagram for the literature search process is provided in Fig. 1. The dataset included articles from 106 journals. Fifty-four clinical journals and 44 technical journals were included. Assigned journal categories are provided in Supplementary Table 1. The distributions of article publication year, body system and modality for are provided in Fig. 2.

### Title, abstract and introduction

**Fig. 3:**
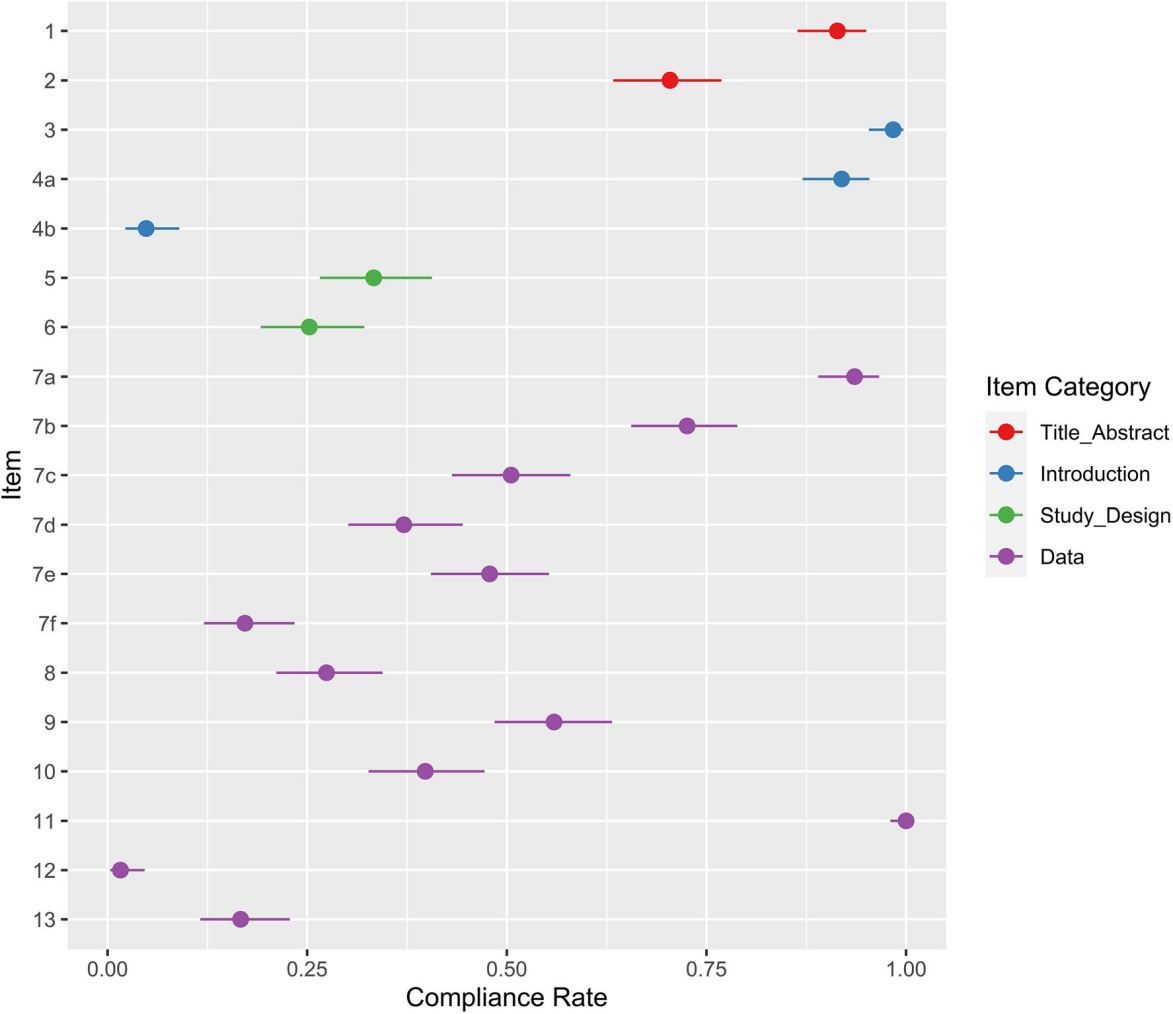
Compliance with CLAIM items 1–13. Compliance rate is defined as the proportion of articles subject to that item which satisfy it. Exemptions are provided in Table 1. Point estimates and 95% confidence intervals are reported

Compliance for items 1–13 is shown in Fig. 3. Ninety-one percent of studies identified their model as a convolutional neural network (170/186, 95% CI 86–95%) and 70% presented a structured abstract (131/186, 95% CI 63–77%). Ninety-eight percent included scientific and clinical background (183/186, 95% CI 95–100%). Although 92% of studies stated objectives (171/186, 95% CI 87–95%), only 4% included explicit hypotheses (8/186, 95% CI 2–8%).

### Study design

Thirty-three percent of studies documented a retrospective or prospective timeframe (62/186, 95% CI 27–41%). Of these, 87% were retrospective (54/62, 95% CI 81–95%), 10% were prospective (6/62, 95% CI 3–18%) and 3% used both retrospective and prospective data (2/62, 95% CI 0–11%). Twenty-five percent of studies specified a goal (47/186, 95% CI 19–32%).

### Data

Ninety-four percent of studies documented their data sources (174/186, 95% CI 89–97%). Of these, 45% used publicly available datasets only (79/174, 95% CI 38–53%), 49% used local data only (85/174, 95% CI 41–57%) and 6% combined public datasets with locally collected data (10/174, 95% CI 0–14%). Seventy-two percent of studies documented the centres from which the data was sourced (134/185, 95% CI 65–79%). Of these, 50% used data from multiple centres (67/135, 95% CI 41–58%). Fifty-one percent of studies detailed the imaging equipment used (94/186, 95% CI 43–58%). Of these, 53% employed equipment from multiple vendors (50/94, 95% CI 43–64%). Image acquisition parameters were documented in 37% of studies (69/186, 95% CI 30–44%). Amongst studies which collected local data, 83% documented institutional review board approval (79/95, 95% CI 74–90%) and 26% documented participant consent (25/95, 95% CI 18–36%). In studies of publicly available data, 9% documented institutional review board approval (7/79, 95% CI 4–17%) and 8% documented participant consent (6/79, 95% CI 3–16%). Twenty-seven percent of studies documented eligibility criteria for their data (50/186, 95% CI 21–34%).

Pre-processing was documented in 69% of studies (128/186, 95% CI 62–75%), though only 53% provided a reproducible methodology (98/186, 95% CI 46–60%). Data subsetting was applied in 42% of studies (78/186, 95% CI 35–49%), of which 95% included methods (74/78, 95% CI 87–99%). As per our inclusion criteria, all studies employed convolutional neural network models, which define predictor features autonomously. We also required an outcome in the domain of radiological cancer diagnosis. Therefore, relevant data elements were defined in 100% of included studies (186/186, 95% CI 98–100%). Nineteen percent of studies performing local data collection documented data anonymisation (18/95, 95% CI 12–28%), though only 3% detailed the methodology (3/95, 95% CI 0–11%). Eighty-four percent of studies performing local data collection documented data anonymisation, institutional review board approval or both (80/95, 95% CI 75–91%). Three percent of studies of publicly available data documented data anonymisation (3/91, 95% CI 1–9%), and none detailed the methodology. Missing data procedures were documented in 17% of studies (31/186, 95% CI 12–23%). Case exclusion was the only strategy employed to manage missing data in these studies (31/31, 95% CI 89–100%).

### Ground truth

**Fig. 4:**
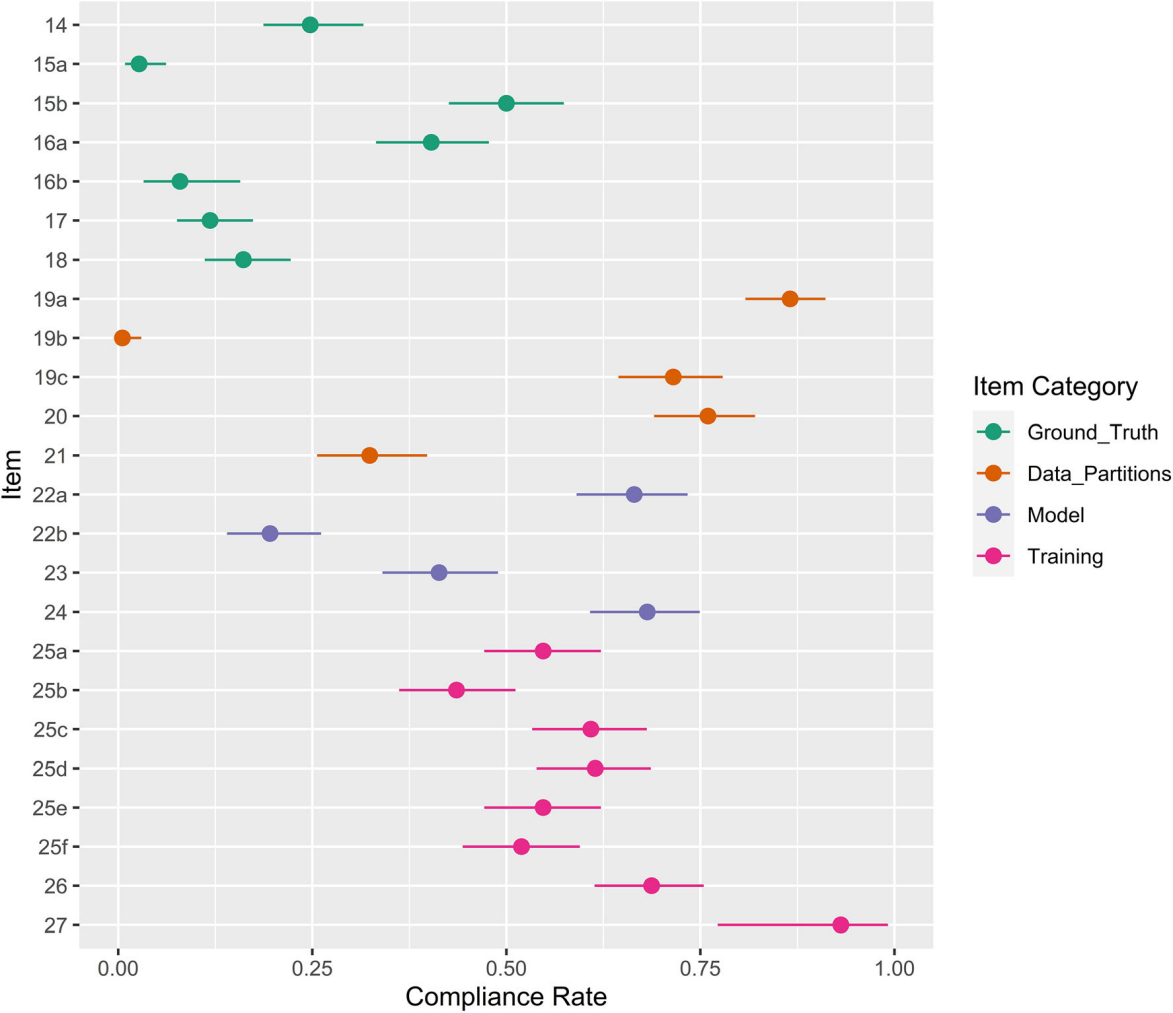
Compliance with CLAIM items 14–27. Compliance rate is defined as the proportion of articles subject to that item which satisfy it. Exemptions are provided in Table 1. Point estimates and 95% confidence intervals are reported

Compliance for items 14–27 is shown in Fig. 4. Twenty-five percent of studies defined the reference standard or used a Common Data Element such as the American College of Radiology Reporting and Data Systems (46/186, 95% CI 19–32%). Three percent of studies provided rationale for the reference standard (5/186, 95% CI 1–6%). However, 50% employed definitive clinical standards such as histopathology, case follow-up or definitive imaging modalities (93/186, 95% CI 43–57%). Of these, 77% used histopathology (72/93, 95% CI 70–86%), 15% used histopathology in combination with follow-up imaging (14/93, 95% CI 8–23%) and 2% used histopathology in combination with definitive imaging (2/93, 95% CI 0–10%). Four percent used follow-up only (14/93, 95% CI 8–23%), and 1% used definitive imaging only (1/93, 95% CI 0–9%).

Forty percent of studies documented image annotation by a radiologist with relevant subspecialist expertise (75/186, 95% CI 33–48%). A further 32% documented annotation by a radiologist with unspecified expertise (60/186, 95% CI 25–40%) and 4% used other clinicians (8/186, 95% CI 0–12%). Of the studies which utilised histopathological ground truth, 8% specified annotation by a pathologist with relevant subspecialist experience (7/88, 95% CI 3–16%). Twelve percent of studies documented the software tools used for image annotation (22/186, 95% CI 8–17%). Eighteen percent of studies provided inter-rater or intra-rater variability statistics (34/186, 95% CI 13–25%), and 27% provided their aggregation strategy (50/186, 95% CI 21–34%), though only 16% provided both (30/186, 95% CI 11–22%).

### Data partitions

Eighty-seven percent of studies reported the number of images modelled (161/186, 95% CI 81–91%), though only 1% provided a power calculation (1/186, 95% CI 0–3%). Seventy-two percent specified the number of study participants in their dataset (133/186, 95% CI 64–78%). Of these, a median of 367 participants were included (IQR 172–1000). Seven studies served only to validate existing models and were exempted from criteria pertaining to model development and data partitioning. Seventy-six percent of modelling studies defined data partitions and their proportions (136/179, 95% CI 69–82%), though 32% specified the level of partition disjunction (58/179, 95% CI 26–40%).

### Model

Sixty-six percent of modelling studies provided a detailed model description (119/179, 95% CI 59–73%) and 20% of modelling studies provided access to source code (35/179, 95% CI 14–26%). Sixty-eight percent documented the development software (122/179, 95% CI 61–75%), though only 41% included the software version (74/179, 95% CI 34–49%). Sixty-eight percent of modelling studies reported model initialisation parameters (122/179, 95% CI 61–75%). Of these, 52% employed transfer learning (93/179, 95% CI 44–59%) and 3% compared transfer learning with random initialisation (4/124, 95% CI 1–8%).

### Training

Sixty-five percent of modelling studies reported data augmentation (117/179, 95% CI 58–72%), though only 54% documented reproducible methodology (96/179, 95% CI 46–61%). Sixty-one percent of modelling studies documented the optimisation algorithm (109/179, 95% CI 53–68%), 61% documented learning rate (110/179, 95% CI 54–69%), 44% documented loss function (78/179, 95% CI 36–51%) and 52% documented batch size (93/179, 95% CI 44–59%). Model selection strategies were documented in 69% of modelling studies (123/179, 95% CI 61–75%). Of 30 studies which employed model ensembling, 93% reported their aggregation methodology (28/30, 95% CI 78–99%).

### Evaluation

**Fig. 5:**
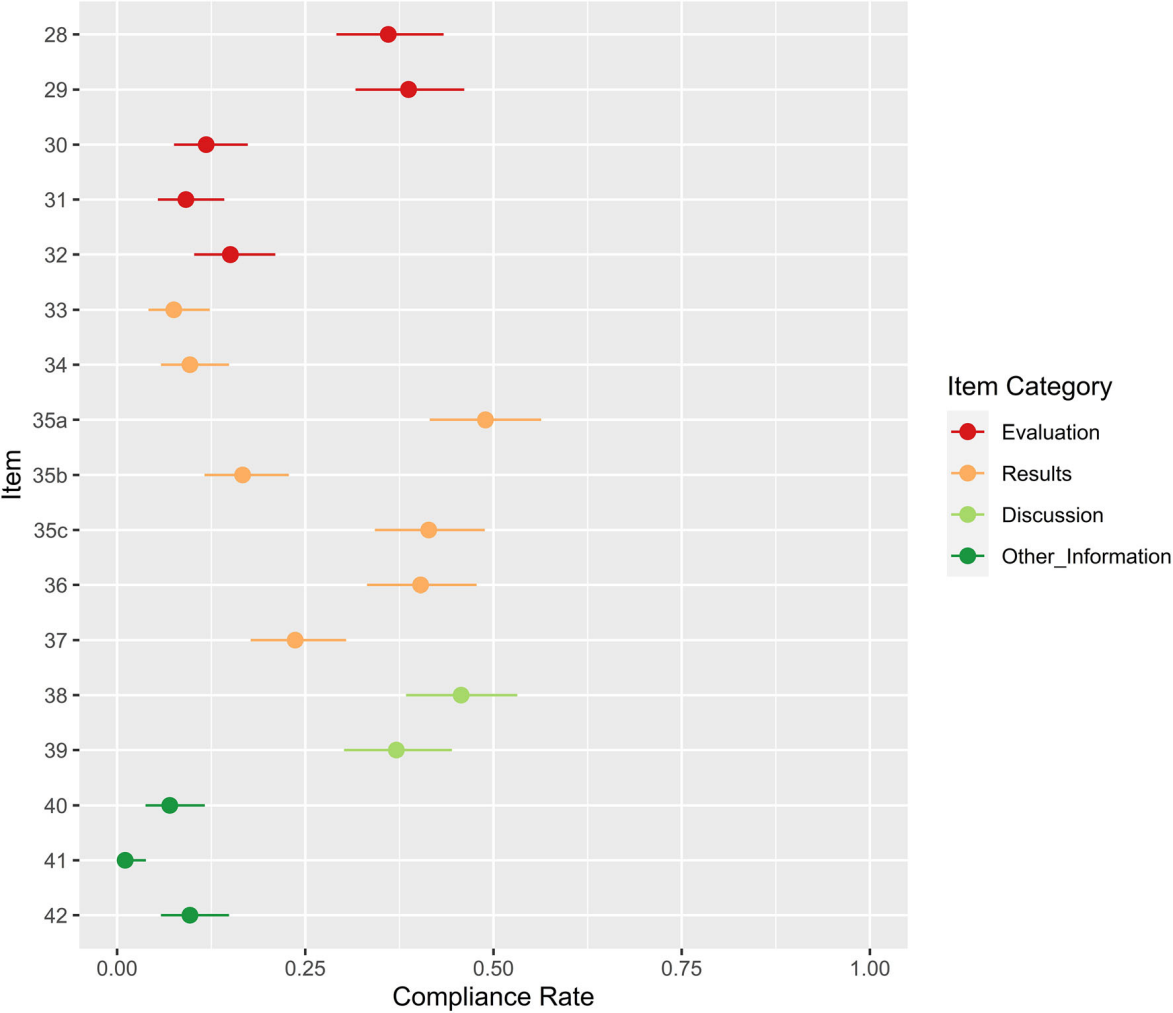
Compliance with CLAIM items 28–42. Compliance rate is defined as the proportion of articles subject to that item which satisfy it. Exemptions are provided in Table 1. Point estimates and 95% confidence intervals are reported

Compliance with CLAIM items 28–42 is shown in Fig. 5. Fifty-five percent of studies defined performance metrics (103/186, 95% CI 48–63%) and 36% provided some rationale for these (67/186, 95% CI 29–43%). Statistical significance of results was reported with methodology in 61% of studies (114/186, 95% CI 54–68%) and without methodology in 97% (181/186, 95% CI 94–99%). Twelve percent of studies evaluated model robustness (22/186, 95% CI 8–17%). Although 25% of studies attempted some interpretation of the model (47/186, 95% CI 19–32%), only 9% provided validated methodology for their procedure (17/186, 95% CI 5–14%).

Forty-nine percent of studies assessed model performance on test data which was explicitly disjunct from training and validation data (91/186, 95% CI 42–57%). Forty-five percent of studies mentioned only two data partitions (83/186, 95% CI 38–53%); their reported results may have represented validation rather than test performance. A further 6% failed to document any data partitions (12/186, 95% CI 0–14%); their reported results may have represented training performance. Forty-one percent of studies benchmarked models against other computational methods (77/186, 95% CI 34–49%). Seventeen percent of studies benchmarked their model against radiologists with relevant subspecialist expertise (31/186, 95% CI 11–23%). Five percent of studies benchmarked their model against radiologists without specifying expertise (9/186, 95% CI 0–11%) and 1% employed other clinicians (1/186, 95% CI 0–7%).

### Results

Case flow diagrams were provided in 8% of studies (14/186, 95% CI 4–12%). Thirty-one percent of studies reported demographic and clinical characteristics of their population (58/186, 95% CI 25–38%). However, only 10% described separate distributions for each data partition (18/186, 95% CI 6–15%). Fifteen percent of studies reported performance metrics on test data from another institution (28/186, 95% CI 8–23%). Thirty-four percent used test data from the same institution (63/186, 95% CI 27–41%). Diagnostic accuracy was reported with confidence intervals in 40% of studies (75/186, 95% CI 33–48%). Twenty-four percent of studies discussed misclassified examples (44/186, 95% CI 18–30%).

### Discussion

Forty-six percent of studies discussed limitations (85/186, 95% CI 38–53%) and 37% clinical implications of their findings (69/186, 95% CI 30–44%).

### Other information

Study registration numbers were provided in 7% of studies (13/186, 95% CI 3.8–11.7%), and study protocols in 1% (2/186, 95% CI 0.1–3.8%). Funding was documented in 65% of studies (121/186, 95% CI 57.7–71.9%) though only 3% included the role of the funding institution (6/186, 95% CI 0.0–10.9%). A further 6% of studies stated that they did not receive funding (12/186, 95% CI 0.0–14.1%). Compliance for items 1–42 is provided in Supplementary Table 3.

### Overall CLAIM compliance

**Fig. 6:**
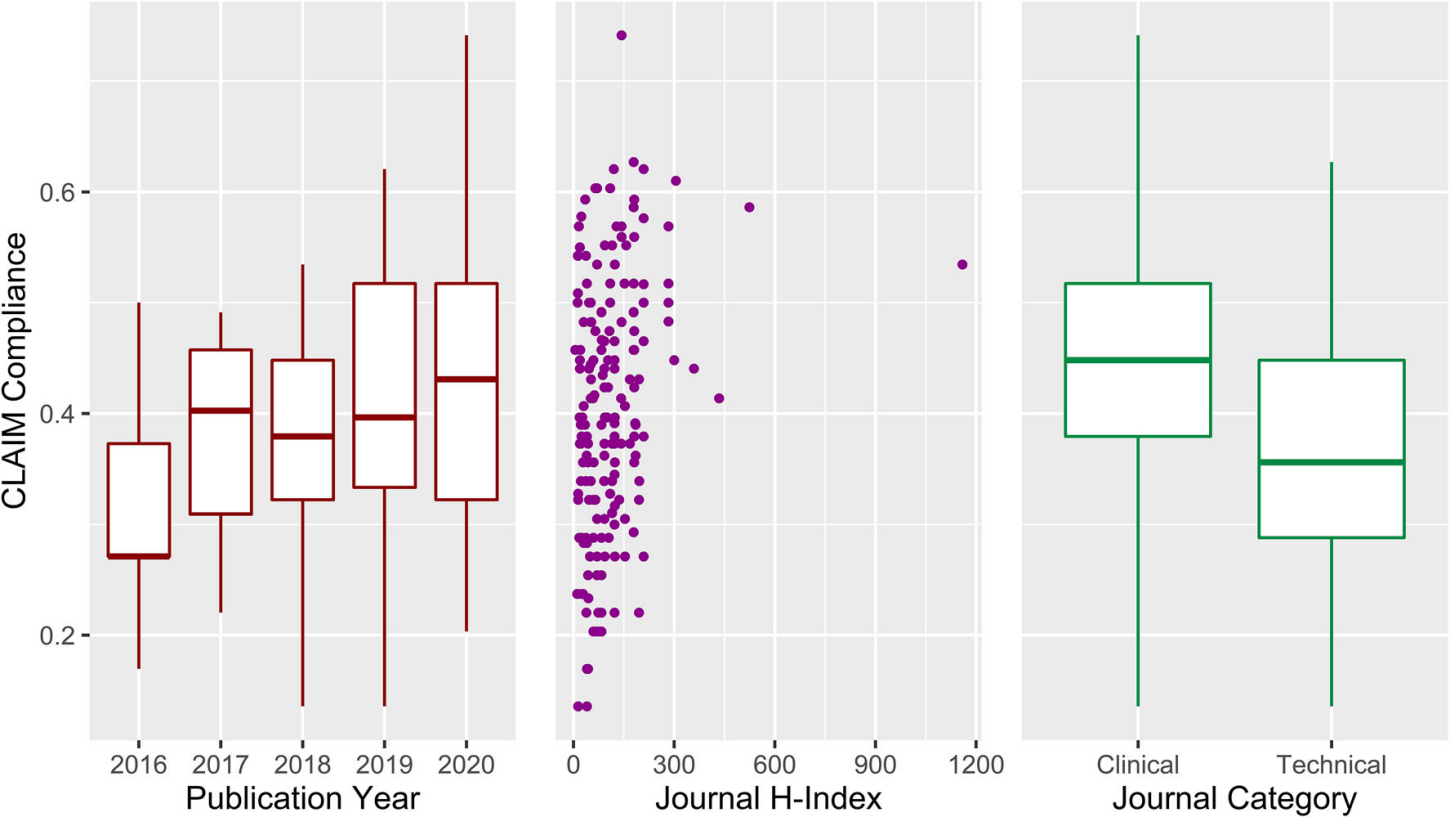
Left: CLAIM compliance over time. Compliance was defined per article by the proportion of applicable items satisfied. Boxplot centrelines indicate median annual compliance. Hinges indicate first and third quartiles. Whiskers indicate maxima and minima. Middle: CLAIM compliance and journal H-index for each article. Right: CLAIM compliance in clinical journals and technical journals. Journals were categorised as either “clinical” or “technical” according to the journal name—names containing any term related to computer science, artificial intelligence or machine learning were assigned the “technical” category. The remaining journals were assigned the “clinical” category

Median CLAIM compliance was 0.40 (IQR 0.33–0.49). Compliance correlated positively with publication year (*ρ* = 0.15, *p* = .04) and journal H-index (*ρ* = 0.27, *p* < .001). Clinical journals demonstrated higher mean compliance than technical journals (0.44 vs. 0.37, *p* < .001). Compliance distribution is visualised with respect to publication year, journal H-index and journal category in Fig. 6.

## Discussion

Radiological AI is undergoing a development phase, reflected in growing annual publication volume and recognition by clinical researchers [33–37]. To safely harness the potential of new methodologies, clinicians have called for realistic, reproducible and ethical research practices [1, 38–44]. The CLAIM guidance sets stringent standards for research in this domain, amalgamating the technical requirements of the statistical learning field [9, 45] with the practicalities of clinical research [1, 2, 15, 46]. We observed improvements in documentation standards improved over time, a finding concurrent with previous reviews of AI research [43, 45]. Compliance was highest in impactful clinical journals, demonstrating the value of design and reporting practices at peer review.

A key opportunity for improvement is model testing, addressed by items 20, 21, 32 and 35. Documentation should specify three disjoint data partitions for CNN modelling (which may be resampled with cross-validation or bootstrapping). Training data is used for model learning, validation data for model selection and test data to assess performance of a finalised model [47, 48]. Half of studies documented two or less partitions—in these cases, results may have represented validation or even training performance. Where data partitions were not disjoint on per-patient basis, data leakage may have occurred despite partitioned model testing. These scenarios bias generalisability metrics optimistically. Some multi-centre studies partitioned data at the patient level rather than the institutional level, missing an opportunity to evaluate inter-institution generalisability.

Evidently, CLAIM has also introduced requirements which depart from current norms. Few studies satisfied item 12, which requires the documentation of data anonymisation methods, an issue which has developed with image recognition capabilities [41, 49, 50]. This requirement may have previously been relaxed for studies of publicly available data or those which documented institutional review board approval, as either case suggests previous certification of data governance procedures. The spirit of the CLAIM guidance is obviation of such assumptions with clear documentation, promoting a culture of research transparency. In many such cases, the burden of improved compliance is minimal, mandating only the documentation of additional information.

Our findings concur with previous reviews of design and reporting standards in both clinical and general-purpose AI research. A review of studies benchmarking AI against radiologists identified deficient documentation of data availability, source code, eligibility and study setting [38]. Reviews of TRIPOD adherence in multivariate diagnostic modelling found deficient model assessment and data description [12, 51, 52]. Reviews of reproducibility in AI research have reported insufficient documentation of data availability, source code, protocols and study registration [43, 45, 53]. Many commentators have advocated for transparency in clinical AI research [19, 38, 40, 42, 43, 53, 54].

We note several limitations to this systematic review. First, as scope was limited to studies published in English, findings were susceptible to language bias. Second, although reporting standards were directly measurable, items relating to study design were only measurable if reported. Consequently, design compliance may have been underestimated in poorly reported studies. This is a general limitation of reviews in this field. Third, articles were read sequentially and therefore readers were potentially susceptible to anchoring bias. The effect of anchoring on the trend and subgroup analyses was minimised by randomisation of the reading order.

## Conclusions

Design and reporting standards have improved in CNN research for radiological cancer diagnosis, though many opportunities remain for further progress. The CLAIM guidance sets a high standard for this developing field, consolidating clinical and technical research requirements to enhance the quality of evidence. Our data supports the need for integration of CLAIM guidance into the design and reporting of CNN studies for radiological cancer diagnosis.

## Supplementary Information

ESM 1 (DOCX 89 kb)

## Abbreviations

AI: Artificial intelligence
CLAIM: Checklist for AI in Medical Imaging
CNN: Convolutional neural network
CONSORT: Consolidated Standards of Reporting Trials
EQUATOR: Enhancing the Quality and Transparency Of health Research
IQR: Interquartile range
MI-CLAIM: Minimum Information about Clinical Artificial Intelligence Modelling
PRISMA: Preferred Reporting Items for Systematic reviews and Meta-Analyses
QUADAS: Quality Assessment Tool for Diagnostic Accuracy Studies
SPIRIT-AI: Standard Protocol Items: Recommendations for Interventional Trials-Artificial Intelligence extension
STARD: Standards for Reporting of Diagnostic Accuracy Studies
STARD-AI: Standards for Reporting of Diagnostic Accuracy Studies - Artificial Intelligence extension
STROBE: Reporting of Observational studies in Epidemiology

## Declarations

### Guarantor

The scientific guarantor of this publication is Vicky Goh (Vicky.goh@kcl.ac.uk).

### Statistics and biometry

No complex statistical methods were necessary for this paper.

### Methodology

• Retrospective

• Multicentre study
