# Supplementary material for: Systematic review of research design and reporting of imaging studies applying convolutional neural networks for radiological cancer diagnosis
Source: Eur Radiol. 2021 Apr 16;31(10):7969–83. doi: 10.1007/s00330-021-07881-2 (PMC8452579; doi:10.1007/s00330-021-07881-2)
Supplement: Supplementary file 1 — (DOCX 89 kb) [file 330_2021_7881_MOESM1_ESM.docx]

**ELECTRONIC SUPPLEMENTARY MATERIAL**

**Search strategy:**

Detail are presented below.

**Search date:**

14/08/20

**Search time interval:**

01/01/2016 – 01/08/2020

**Searched databases:**

Medline, embase, pubmed and scopus

**Pubmed query:**

(ct[Title/Abstract] OR computed tomography[Title/Abstract] OR mri[Title/Abstract] OR magnetic resonance imaging[Title/Abstract] OR ultraso*[Title/Abstract] OR sonography[Title/Abstract] OR xray[Title/Abstract] OR x-ray[Title/Abstract] OR radiograph*[Title/Abstract] OR medical imag*[Title/Abstract] OR radiolo*[Title/Abstract] OR pet[Title/Abstract] OR positron emission tomography[Title/Abstract] OR spect[Title/Abstract] OR single photon emission computed tomography[Title/Abstract] OR mammograph*)

NOT (fMRI[Title/Abstract])

AND (deep learning[Title/Abstract] OR convolutional neural network*[Title/Abstract] OR cnn[Title/Abstract] OR convnet*[Title/Abstract] OR gan[Title/Abstract] OR adversarial network*[Title/Abstract])

AND (diagnos*[Title/Abstract])

AND (cancer[Title/Abstract] OR tumor*[Title/Abstract] OR tumour*[Title/Abstract] OR nodule*[Title/Abstract] OR benign[Title/Abstract] OR malignan*[Title/Abstract] OR metasta*[Title/Abstract] OR carcino*[Title/Abstract] OR lymphoma*[Title/Abstract] OR melano*[Title/Abstract] OR sarcoma*[Title/Abstract] OR dysplas*[Title/Abstract] OR metasta*[Title/Abstract] OR leukaemia*[Title/Abstract])

NOT (grading[Title/Abstract] OR prognos*[Title/Abstract] OR genotyp*[Title/Abstract] OR subtyp*[Title/Abstract])

NOT (segmentation[Title/Abstract] OR reconstruction[Title/Abstract] OR registration[Title/Abstract] OR noise[Title/Abstract] OR resolution[Title/Abstract] OR enhanc*[Title/Abstract] OR landmark[Title/Abstract])

NOT (proteomic*[Title/Abstract] OR metabolomic*[Title/Abstract] or genomic*[Title/Abstract])

NOT (dog[Title/Abstract] OR dogs[Title/Abstract] OR canine[Title/Abstract] OR cat[Title/Abstract] OR cats[Title/Abstract] OR feline[Title/Abstract] OR cow[Title/Abstract] OR cattle[Title/Abstract] OR bovine[Title/Abstract] OR sheep[Title/Abstract] OR ovine[Title/Abstract] OR pig OR veterinary[Title/Abstract] OR animal[Title/Abstract])

NOT (cost-effectiv*[Title/Abstract] OR cost-minimi*[Title/Abstract] OR cost-benefit[Title/Abstract])

NOT (review[Title/Abstract] OR introduct*[Title/Abstract] OR primer[Title/Abstract] OR case report[Title/Abstract] OR survey[Title/Abstract] OR questionnaire[Title/Abstract] OR editorial[Title/Abstract])

AND ((humans[Filter])

AND (english[Filter]))

2016/01:2020/08 [edat]

**Ovid query:**

Databases: Embase and Ovid MEDLINE (R)

(ct OR computed tomography OR mri OR magnetic resonance imaging OR ultraso* OR sonography OR xray OR x-ray OR radiograph* OR medical imag* OR radiolo* OR pet OR positron emission tomography OR spect OR single photon emission computed tomography OR mammograph* NOT fmri).ti,ab.

AND (deep learning OR cnn OR convolutional neural network* OR convnet* OR gan OR adversarial network* OR transfer learning).ti,ab.

AND (diagnos*).ti,ab.

AND (cancer OR tumor* OR tumour* OR nodule* OR benign OR malignan* OR metasta* OR carcino* OR lymphoma* OR melano* OR sarcoma* OR dysplas* OR metasta* OR leukaemia*).ti,ab.

NOT (grading OR prognos* OR genotyp* OR subtyp*).ti,ab.

NOT (segmentation OR reconstruction OR registration OR noise OR resolution OR enhanc* OR landmark).ti,ab.

NOT (proteomic* OR metabolomic* or genomic*).ti,ab.

NOT (dog OR dogs OR canine OR cat OR cats OR feline OR cow OR cattle OR bovine OR sheep OR ovine OR pig OR porcine OR veterinary OR animal).ti,ab.

NOT (review OR introduct* OR primer OR case report OR conference OR survey OR questionnaire OR editorial).af.

NOT (cost-effectiv* OR cost-minimi* OR cost-benefit).ti.

Filters: English Language, Humans, Publication Year 2016-2020

**Scopus query:**

(

TITLE-ABS-KEY(

(ct OR "computed tomography" OR mri OR "magnetic resonance imaging" OR ultraso* OR sonography* OR xray OR "x-ray" OR radiograph* OR "medical imag*" OR radiolo* OR pet OR "positron emission tomography" OR spect OR "single photon emission computed tomography" OR mammograph*)

AND (“deep learning” OR “convolutional neural network*” OR “cnn” OR “convnet*” OR “gan” OR “adversarial network*”)

AND (diagnos*)

AND (cancer OR tumor* OR tumour* OR nodule* OR benign OR malignan* OR metasta* OR carcino* OR lymphoma* OR melano* OR sarcoma* OR dysplas* OR metasta* or leukaemia*)

AND NOT (

(fMRI)

OR (grading OR prognos* OR genotyp* OR “bone age”)

OR (segment* OR reconstruct* OR regist* OR noise OR resolution OR enhanc* or landmark*)

OR (dog OR dogs OR canine OR cat OR cats OR feline OR cow OR cattle OR bovine OR sheep OR ovine OR pig OR porcine OR veterinary OR animal)

OR (proteomic* OR metabolomic* or genomic*)

OR (review OR introduct* OR primer OR “case report” OR survey OR questionnaire)

)

)

AND PUBYEAR > 2015

AND ( LIMIT-TO ( SRCTYPE,"j" ) )

AND ( LIMIT-TO ( DOCTYPE,"ar" ) )

AND ( LIMIT-TO ( LANGUAGE,"English" ) )

AND NOT (

TITLE (“cost-effectiv*” OR “cost-minimi*” OR “cost-benefit” or economic)

OR SUBJAREA(PHAR OR DENT OR MATE OR PSYC OR CENG OR CHEM OR ENVI OR SOCI OR ARTS OR ECON OR BIOC OR AGRI OR BUSI OR EART OR ENER OR PHYS OR ARTS OR VETE OR IMMU)

)

)

**Supplementary Table 1. Assigned journal categories and number of included articles.**

| **Title** | **Journal** | **Authors** | **Year** | **Suitability** |
| --- | --- | --- | --- | --- |
| 18F-FDG PET/CT uptake classification in lymphoma and lung cancer by using deep convolutional neural networks. | Radiology | Sibille L., Seifert R., Avramovic N., Vehren T., Spottiswoode B., Zuehlsdorff S., Schafers M. | 2020 | Included in analysis |
| 3D deep learning for detecting pulmonary nodules in CT scans | Journal of the American Medical Informatics Association | Gruetzemacher R., Gupta A., Paradice D. | 2018 | Included in analysis |
| A 3D Probabilistic Deep Learning System for Detection and Diagnosis of Lung Cancer Using Low-Dose CT Scans. | IEEE Transactions on Medical Imaging | Ozdemir O., Russell R.L., Berlin A.A. | 2020 | Included in analysis |
| A CNN based breast tumor classifier using mendeley BUS dataset | International Journal of Innovative Technology and Exploring Engineering | Kameswari S.S.D., Kameswari S.S.D., Vijayakumar V. | 2019 | Included in analysis |
| A deep 3D residual CNN for false-positive reduction in pulmonary nodule detection. | Medical Physics | Jin H, Li Z, Tong R, Lin L | 2018 | Included in analysis |
| A deep feature based framework for breast masses classification | Neurocomputing | Jiao Z., Gao X., Wang Y., Li J. | 2016 | Included in analysis |
| A deep learning framework for supporting the classification of breast lesions in ultrasound images. | Physics in medicine and biology | Han S., Kang H.-K., Jeong J.-Y., Park M.-H., Kim W., Bang W.-C., Seong Y.-K. | 2017 | Included in analysis |
| A Deep Learning-Based Framework for Automatic Brain Tumors Classification Using Transfer Learning | Circuits, Systems, and Signal Processing | Rehman A., Naz S., Razzak M.I., Akram F., Imran M. | 2020 | Included in analysis |
| A deep stacked random vector functional link network autoencoder for diagnosis of brain abnormalities and breast cancer. | Biomedical Signal Processing and Control | Nayak D.R., Dash R., Majhi B., Pachori R.B., Zhang Y. | 2020 | Included in analysis |
| A fundamental study assessing the diagnostic performance of deep learning for a brain metastasis detection task | Magnetic Resonance in Medical Sciences | Noguchi T., Uchiyama F., Kawata Y., Machitori A., Shida Y., Okafuji T., Yokoyama K., Inaba Y., Tajima T. | 2020 | Included in analysis |
| A knowledge-driven feature learning and integration method for breast cancer diagnosis on multi-sequence MRI. | Magnetic Resonance Imaging | Feng H., Cao J., Wang H., Xie Y., Yang D., Feng J., Chen B. | 2020 | Included in analysis |
| A Lightweight Multi-Section CNN for Lung Nodule Classification and Malignancy Estimation. | IEEE Journal of Biomedical and Health Informatics | Sahu P., Yu D., Dasari M., Hou F., Qin H. | 2019 | Included in analysis |
| A Novel Computer-Aided Diagnosis Scheme on Small Annotated Set: G2C-CAD. | BioMed Research International | Zheng G., Han G., Soomro N.Q., Ma L., Zhang F., Zhao Y., Zhao X., Zhou C. | 2019 | Included in analysis |
| A novel diagnostic method for pituitary adenoma based on magnetic resonance imaging using a convolutional neural network. | Pituitary | Qian Y., Qiu Y., Li C.-C., Wang Z.-Y., Cao B.-W., Huang H.-X., Ni Y.-H., Chen L.-L., Sun J.-Y. | 2020 | Included in analysis |
| A Novel Hybrid Feature Extraction Model for Classification on Pulmonary Nodules. | Asian Pacific Journal of Cancer Prevention: Apjcp | Kailasam SP, Sathik MM | 2019 | Included in analysis |
| A Novel Liver Image Classification Method Using Perceptual Hash-Based Convolutional Neural Network | Arabian Journal for Science and Engineering | Ã–zyurt F., Tuncer T., Avci E., KoÃ§ M., SerhatlioÄŸlu Ä°. | 2019 | Included in analysis |
| A Novel Method for Classifying Liver and Brain Tumors Using Convolutional Neural Networks, Discrete Wavelet Transform and Long Short-Term Memory Networks. | Sensors (Basel, Switzerland) | Kutlu H., Avci E. | 2019 | Included in analysis |
| A parasitic metric learning net for breast mass classification based on mammography | Pattern Recognition | Jiao Z., Gao X., Wang Y., Li J. | 2018 | Included in analysis |
| A pre-trained convolutional neural network based method for thyroid nodule diagnosis. | Ultrasonics | Ma J., Wu F., Zhu J., Xu D., Kong D. | 2017 | Included in analysis |
| A robust convolutional neural network for lung nodule detection in the presence of foreign bodies. | Scientific reports | Schultheiss M., Schober S.A., Lodde M., Bodden J., Aichele J., Muller-Leisse C., Renger B., Pfeiffer F., Pfeiffer D. | 2020 | Included in analysis |
| A semi-automated annotation algorithm based on weakly supervised learning for medical images. | Biocybernetics and Biomedical Engineering | Li H., Zhang B., Zhang Y., Liu W., Mao Y., Huang J., Wei L. | 2020 | Included in analysis |
| Agile convolutional neural network for pulmonary nodule classification using CT images. | International Journal of Computer Assisted Radiology & Surgery | Zhao X, Liu L, Qi S, Teng Y, Li J, Qian W | 2018 | Included in analysis |
| An early-stage classification of lung nodules by an android based application using deep convolution neural network with cost-sensitive loss function and progressive scaling approach | International Journal of Advanced Trends in Computer Science and Engineering | Pandya M.D., Jardosh S., Thakkar A.R. | 2020 | Included in analysis |
| An inception module CNN classifiers fusion method on pulmonary nodule diagnosis by signs | Tsinghua Science and Technology | Zheng G., Han G., Soomro N.Q. | 2020 | Included in analysis |
| An interpretable deep hierarchical semantic convolutional neural network for lung nodule malignancy classification | Expert Systems with Applications | Shen S., Han S.X., Aberle D.R., Bui A.A., Hsu W. | 2019 | Included in analysis |
| An investigation of the classification accuracy of a deep learning framework-based computer-aided diagnosis system in different pathological types of breast lesions. | Journal of Thoracic Disease | Xiao M., Zhao C., Zhu Q., Zhang J., Liu H., Li J., Jiang Y. | 2019 | Included in analysis |
| Application of convolutional neural network in the diagnosis of Jaw tumors | Healthcare Informatics Research | Poedjiastoeti W., Suebnukarn S. | 2018 | Included in analysis |
| Application of deep learning to the diagnosis of cervical lymph node metastasis from thyroid cancer with CT: external validation and clinical utility for resident training. | European Radiology | Lee J.H., Ha E.J., Kim D.Y., Jung Y.J., Heo S., Jang Y.-H., An S.H., Lee K. | 2020 | Included in analysis |
| Applying Data-driven Imaging Biomarker in Mammography for Breast Cancer Screening: Preliminary Study | Scientific Reports | Kim E.-K., Kim H.-E., Han K., Kang B.J., Sohn Y.-M., Woo O.H., Lee C.W. | 2018 | Included in analysis |
| Artificial intelligence system of faster region-based convolutional neural network surpassing senior radiologists in evaluation of metastatic lymph nodes of rectal cancer. | Chinese Medical Journal | Ding L., Liu G.-W., Zhao B.-C., Zhou Y.-P., Li S., Zhang Z.-D., Guo Y.-T., Li A.-Q., Lu Y., Yao H.-W., Yuan W.-T., Wang G.-Y., Zhang D.-L., Wang L. | 2019 | Included in analysis |
| Assessing the Accuracy of a Deep Learning Method to Risk Stratify Indeterminate Pulmonary Nodules. | American journal of respiratory and critical care medicine | Massion P.P., Antic S., Ather S., Arteta C., Brabec J., Chen H., Declerck J., Dufek D., Hickes W., Kadir T., Kunst J., Landman B.A., Munden R.F., Novotny P., Peschl H., Pickup L.C., Santos C., Smith G.T., Talwar A., Gleeson F. | 2020 | Included in analysis |
| Attentive and ensemble 3D dual path networks for pulmonary nodules classification | Neurocomputing | Jiang H., Gao F., Xu X., Huang F., Zhu S. | 2020 | Included in analysis |
| Automated Breast Ultrasound Lesions Detection Using Convolutional Neural Networks. | IEEE Journal of Biomedical and Health Informatics | Yap M.H., Pons G., Marti J., Ganau S., Sentis M., Zwiggelaar R., Davison A.K., Marti R. | 2018 | Included in analysis |
| Automated diagnosis of prostate cancer in multi-parametric MRI based on multimodal convolutional neural networks. | Physics in medicine and biology | Le M.H., Chen J., Wang L., Wang Z., Liu W., Cheng K.-T.T., Yang X. | 2017 | Included in analysis |
| Automated lung cancer diagnosis using three-dimensional convolutional neural networks | Medical and Biological Engineering and Computing | Perez G., Arbelaez P. | 2020 | Included in analysis |
| Automated Lung Nodule Detection and Classification Using Deep Learning Combined with Multiple Strategies. | Sensors (Basel, Switzerland) | Nasrullah N., Sang J., Alam M.S., Mateen M., Cai B., Hu H. | 2019 | Included in analysis |
| Automated mammographic mass detection using deformable convolution and multiscale features. | Medical and Biological Engineering and Computing | Peng J., Bao C., Hu C., Wang X., Jian W., Liu W. | 2020 | Included in analysis |
| Automated Pulmonary Nodule Classification in Computed Tomography Images Using a Deep Convolutional Neural Network Trained by Generative Adversarial Networks. | BioMed Research International | Onishi Y., Teramoto A., Tsujimoto M., Tsukamoto T., Saito K., Toyama H., Imaizumi K., Fujita H. | 2019 | Included in analysis |
| Automated pulmonary nodule detection in CT images using deep convolutional neural networks | Pattern Recognition | Xie H., Yang D., Sun N., Chen Z., Zhang Y. | 2019 | Included in analysis |
| Automatic classification of ultrasound breast lesions using a deep convolutional neural network mimicking human decision-making | European Radiology | Ciritsis A., Rossi C., Eberhard M., Marcon M., Becker A.S., Boss A. | 2019 | Included in analysis |
| Automatic detection and classification of regions of FDG uptake in whole-body PET-CT lymphoma studies | Computerized Medical Imaging and Graphics | Bi L., Kim J., Kumar A., Wen L., Feng D., Fulham M. | 2017 | Included in analysis |
| Automatic diagnosis for thyroid nodules in ultrasound images by deep neural networks. | Medical Image Analysis | Wang L., Zhang L., Zhu M., Qi X., Yi Z. | 2020 | Included in analysis |
| Automatic feature learning using multichannel ROI based on deep structured algorithms for computerized lung cancer diagnosis. | Computers in Biology and Medicine | Sun W., Zheng B., Qian W. | 2017 | Included in analysis |
| Automatic lung cancer detection in low-dose lung CTs using transfer learning | Journal of Advanced Research in Dynamical and Control Systems | Akila Agnes S., Anitha J. | 2018 | Included in analysis |
| Automatic lung nodule detection using a 3D deep convolutional neural network combined with a multi-scale prediction strategy in chest CTs. | Computers in Biology and Medicine | Gu Y., Lu X., Yang L., Zhang B., Yu D., Zhao Y., Gao L., Wu L., Zhou T. | 2018 | Included in analysis |
| Automatic Scoring of Multiple Semantic Attributes with Multi-Task Feature Leverage: A Study on Pulmonary Nodules in CT Images. | IEEE Transactions on Medical Imaging | Chen S., Qin J., Ji X., Lei B., Wang T., Ni D., Cheng J.-Z. | 2017 | Included in analysis |
| Automatic thyroid nodule recognition and diagnosis in ultrasound imaging with the YOLOv2 neural network. | World Journal of Surgical Oncology | Wang L., Yang S., Zhao C., Tian G., Gao Y., Chen Y., Lu Y. | 2019 | Included in analysis |
| Brain tumor classification using Convolutional Neural Networks. | Biomedical and Pharmacology Journal | Seetha J., Raja S.S. | 2018 | Included in analysis |
| Brain tumor classification using deep CNN features via transfer learning. | Computers in Biology and Medicine | Deepak S., Ameer P.M. | 2019 | Included in analysis |
| BrainMRNet: Brain tumor detection using magnetic resonance images with a novel convolutional neural network model | Medical Hypotheses | ToÄŸaÃ§ar M., Ergen B., CÃ¶mert Z. | 2020 | Included in analysis |
| Breast Cancer Classification in Automated Breast Ultrasound Using Multiview Convolutional Neural Network with Transfer Learning. | Ultrasound in Medicine and Biology | Wang Y., Choi E.J., Choi Y., Zhang H., Jin G.Y., Ko S.-B. | 2020 | Included in analysis |
| Breast cancer diagnosis in digital breast tomosynthesis: Effects of training sample size on multi-stage transfer learning using deep neural nets. | IEEE Transactions on Medical Imaging | Samala R.K., Chan H.-P., Hadjiiski L., Helvie M.A., Richter C.D., Cha K.H. | 2019 | Included in analysis |
| Breast cancer diagnosis using thermography and convolutional neural networks. | Medical Hypotheses | Ekici S., Jawzal H. | 2020 | Included in analysis |
| Breast lesion classification based on ultrasonic radio-frequency signals using convolutional neural networks. | Biocybernetics and Biomedical Engineering | Jarosik P., Klimonda Z., Lewandowski M., Byra M. | 2020 | Included in analysis |
| Breast mass detection from the digitized X-ray mammograms based on the combination of deep active learning and self-paced learning | Future Generation Computer Systems | Shen R., Yan K., Tian K., Jiang C., Zhou K. | 2019 | Included in analysis |
| Breast Microcalcification Diagnosis Using Deep Convolutional Neural Network from Digital Mammograms. | Computational and Mathematical Methods in Medicine | Cai H., Huang Q., Rong W., Song Y., Li J., Wang J., Chen J., Li L. | 2019 | Included in analysis |
| Cervical Cancer Detection and Diagnosis Based on Saliency Single Shot MultiBox Detector in Ultrasonic Elastography | J Med Syst | Wei S, Dai P, Wang Z. | 2019 | Included in analysis |
| Classification of benign and malignant lung nodules from CT images based on hybrid features. | Physics in medicine and biology | Zhang G., Yang Z., Gong L., Jiang S., Wang L. | 2019 | Included in analysis |
| Classification of breast cancer in ultrasound imaging using a generic deep learning analysis software: a pilot study. | British Journal of Radiology | BeCkeR A.S., MuelleR M., StOFFel E., MARCOn M., ghAFOOR S., Boss A. | 2018 | Included in analysis |
| Classification of breast masses on ultrasound shear wave elastography using convolutional neural networks. | Ultrasonic imaging | Fujioka T., Katsuta L., Kubota K., Mori M., Kikuchi Y., Kato A., Oda G., Nakagawa T., Kitazume Y., Tateishi U. | 2020 | Included in analysis |
| Classification of lung nodules based on CT images using squeeze-and-excitation network and aggregated residual transformations | Radiologia Medica | Zhang G., Yang Z., Gong L., Jiang S., Wang L., Zhang H. | 2020 | Included in analysis |
| Classification of lung nodules in CT scans using three-dimensional deep convolutional neural networks with a checkpoint ensemble method. | BMC medical imaging | Jung H., Kim B., Lee I., Lee J., Kang J. | 2018 | Included in analysis |
| Classification of Mammogram Images Using Multiscale all Convolutional Neural Network (MA-CNN). | Journal of Medical Systems | Agnes S.A., Anitha J., Pandian S.I.A., Peter J.D. | 2020 | Included in analysis |
| Classifying symmetrical differences and temporal change for the detection of malignant masses in mammography using deep neural networks | Journal of Medical Imaging | Kooi T., Karssemeijer N. | 2017 | Included in analysis |
| Cloud-Based Automated Clinical Decision Support System for Detection and Diagnosis of Lung Cancer in Chest CT. | IEEE Journal of Translational Engineering in Health and Medicine | Masood A., Yang P., Sheng B., Li H., Li P., Qin J., Lanfranchi V., Kim J., Feng D.D. | 2020 | Included in analysis |
| Combination of generative adversarial network and convolutional neural network for automatic subcentimeter pulmonary adenocarcinoma classification. | Quantitative Imaging in Medicine and Surgery | Wang Y., Zhou L., Wang M., Shao C., Shi L., Yang S., Zhang Z., Feng M., Shan F., Liu L. | 2020 | Included in analysis |
| Comparing two classes of end-to-end machine-learning models in lung nodule detection and classification: MTANNs vs. CNNs | Pattern Recognition | Tajbakhsh N., Suzuki K. | 2017 | Included in analysis |
| Computer-Aided Diagnosis (CAD) of Pulmonary Nodule of Thoracic CT Image Using Transfer Learning | Journal of Digital Imaging | Zhang S., Sun F., Wang N., Zhang C., Yu Q., Zhang M., Babyn P., Zhong H. | 2019 | Included in analysis |
| Computer-aided diagnosis of breast ultrasound images using ensemble learning from convolutional neural networks. | Computer Methods and Programs in Biomedicine | Moon W.K., Lee Y.-W., Ke H.-H., Lee S.H., Huang C.-S., Chang R.-F. | 2020 | Included in analysis |
| Computer-aided diagnosis of lung nodule classification between benign nodule, primary lung cancer, and metastatic lung cancer at different image size using deep convolutional neural network with transfer learning. | PLoS ONE | Nishio M., Sugiyama O., Yakami M., Ueno S., Kubo T., Kuroda T., Togashi K. | 2018 | Included in analysis |
| Computer-aided diagnosis of prostate cancer on magnetic resonance imaging using a convolutional neural network algorithm. | BJU International | Ishioka J., Matsuoka Y., Uehara S., Yasuda Y., Kijima T., Yoshida S., Yokoyama M., Saito K., Kihara K., Numao N., Kimura T., Kudo K., Kumazawa I., Fujii Y. | 2018 | Included in analysis |
| Computer-aided diagnosis scheme for determining histological classification of breast lesions on ultrasonographic images using convolutional neural network. | Diagnostics | Hizukuri A., Nakayama R. | 2018 | Included in analysis |
| Computer-aided diagnosis system for breast ultrasound images using deep learning. | Physics in medicine and biology | Tanaka H., Chiu S.-W., Watanabe T., Kaoku S., Yamaguchi T. | 2019 | Included in analysis |
| Computer-aided tumor detection in automated breast ultrasound using a 3-D convolutional neural network. | Computer Methods and Programs in Biomedicine | Moon W.K., Huang Y.-S., Hsu C.-H., Chang Chien T.-Y., Chang J.M., Lee S.H., Huang C.-S., Chang R.-F. | 2020 | Included in analysis |
| Computer-Assisted Decision Support System in Pulmonary Cancer detection and stage classification on CT images. | Journal of Biomedical Informatics | Masood A., Sheng B., Li P., Hou X., Wei X., Qin J., Feng D. | 2018 | Included in analysis |
| Convolutional Neural Network Detection of Axillary Lymph Node Metastasis Using Standard Clinical Breast MRI. | Clinical Breast Cancer | Ren T., Cattell R., Duanmu H., Huang P., Li H., Vanguri R., Liu M.Z., Jambawalikar S., Ha R., Wang F., Cohen J., Bernstein C., Bangiyev L., Duong T.Q. | 2020 | Included in analysis |
| Co-trained convolutional neural networks for automated detection of prostate cancer in multi-parametric MRI. | Medical Image Analysis | Yang X., Liu C., Wang Z., Yang J., Min H.L., Wang L., Cheng K.-T.T. | 2017 | Included in analysis |
| CT evaluation of extranodal extension of cervical lymph node metastases in patients with oral squamous cell carcinoma using deep learning classification. | Oral Radiology | Ariji Y., Sugita Y., Nagao T., Nakayama A., Fukuda M., Kise Y., Nozawa M., Nishiyama M., Katumata A., Ariji E. | 2020 | Included in analysis |
| CT-based deep learning model to differentiate invasive pulmonary adenocarcinomas appearing as subsolid nodules among surgical candidates: comparison of the diagnostic performance with a size-based logistic model and radiologists. | European Radiology | Kim H., Lee D., Cho W.S., Lee J.C., Goo J.M., Kim H.C., Park C.M. | 2020 | Included in analysis |
| Deep convolutional neural network applied to the liver imaging reporting and data system (LI-RADS) version 2014 category classification: a pilot study | Abdominal Radiology | Yamashita R., Mittendorf A., Zhu Z., Fowler K.J., Santillan C.S., Sirlin C.B., Bashir M.R., Do R.K.G. | 2020 | Included in analysis |
| Deep convolutional neural network for the diagnosis of thyroid nodules on ultrasound. | Head and Neck | Ko S.Y., Lee J.H., Yoon J.H., Na H., Hong E., Han K., Jung I., Kim E.-K., Moon H.J., Park V.Y., Lee E., Kwak J.Y. | 2019 | Included in analysis |
| Deep Convolutional Neural Networks for breast cancer screening. | Computer Methods and Programs in Biomedicine | Chougrad H., Zouaki H., Alheyane O. | 2018 | Included in analysis |
| Deep feature-based automatic classification of mammograms. | Medical and Biological Engineering and Computing | Arora R., Rai P.K., Raman B. | 2020 | Included in analysis |
| Deep learning based classification of ultrasound images for thyroid nodules: A large scale of pilot study. | Annals of Translational Medicine | Guan Q., Wang Y., Du J., Qin Y., Lu H., Xiang J., Wang F. | 2019 | Included in analysis |
| Deep learning for automated detection of cyst and tumors of the jaw in panoramic radiographs. | Journal of Clinical Medicine | Yang H., Jo E., Kim H.J., Cha I.-H., Jung Y.-S., Nam W., Kim J.-Y., Kim J.-K., Kim Y.H., Oh T.G., Han S.-S., Kim H., Kim D. | 2020 | Included in analysis |
| Deep learning for breast cancer diagnosis from mammograms â€” A comparative study | Journal of Imaging | Tsochatzidis L., Costaridou L., Pratikakis I. | 2019 | Included in analysis |
| Deep learning for differentiation of benign and malignant solid liver lesions on ultrasonography. | Abdominal Radiology | Xi I.L., Wu J., Guan J., Zhang P.J., Horii S.C., Soulen M.C., Zhang Z., Bai H.X. | 2020 | Included in analysis |
| Deep learning for liver tumor diagnosis part II: convolutional neural network interpretation using radiologic imaging features. | European Radiology | Wang C.J., Hamm C.A., Savic L.J., Ferrante M., Schobert I., Schlachter T., Lin M.D., Weinreb J.C., Duncan J.S., Chapiro J., Letzen B. | 2019 | Included in analysis |
| Deep learning for lung Cancer detection and classification | Multimedia Tools and Applications | Asuntha A., Srinivasan A. | 2020 | Included in analysis |
| Deep learning in ct images: Automated pulmonary nodule detection for subsequent management using convolutional neural network | Cancer Management and Research | Xu Y.-M., Zhang T., Xu H., Qi L., Zhang W., Zhang Y.-D., Gao D.-S., Yuan M., Yu T.-F. | 2020 | Included in analysis |
| Deep Learning to Classify Intraductal Papillary Mucinous Neoplasms Using Magnetic Resonance Imaging. | Pancreas | Corral J.E., Hussein S., Kandel P., Bolan C.W., Bagci U., Wallace M.B. | 2019 | Included in analysis |
| Deep Learning-based Automatic Detection Algorithm for Reducing Overlooked Lung Cancers on Chest Radiographs. | Radiology | Jang S., Song H., Shin Y.J., Kim J., Lee K.W., Lee S.S., Lee W., Lee S., Lee K.H. | 2020 | Included in analysis |
| Deep neural network with generative adversarial networks pre-training for brain tumor classification based on MR images | Biomedical Signal Processing and Control | Ghassemi N., Shoeibi A., Rouhani M. | 2020 | Included in analysis |
| Deep transfer learning-based prostate cancer classification using 3 Tesla multi-parametric MRI | Abdominal Radiology | Zhong X., Cao R., Shakeri S., Scalzo F., Lee Y., Enzmann D.R., Wu H.H., Raman S.S., Sung K. | 2019 | Included in analysis |
| Deep-Learning Detection of Cancer Metastases to the Brain on MRI. | Journal of Magnetic Resonance Imaging | Zhang M., Young G.S., Chen H., Qin L., McFaline-Figueroa J.R., Reardon D.A., Cao X., Wu X., Xu X. | 2020 | Included in analysis |
| DeepLesion: Automated mining of large-scale lesion annotations and universal lesion detection with deep learning | Journal of Medical Imaging | Yan K., Wang X., Lu L., Summers R.M. | 2018 | Included in analysis |
| Deeply-Supervised Networks with Threshold Loss for Cancer Detection in Automated Breast Ultrasound | IEEE Transactions on Medical Imaging | Wang Y., Wang N., Xu M., Yu J., Qin C., Luo X., Yang X., Wang T., Li A., Ni D. | 2020 | Included in analysis |
| Detecting and classifying lesions in mammograms with Deep Learning | Scientific Reports | Ribli D., HorvÃ¡th A., Unger Z., Pollner P., Csabai I. | 2018 | Included in analysis |
| Detecting mammographically occult cancer in women with dense breasts using deep convolutional neural network and Radon Cumulative Distribution Transform | Journal of Medical Imaging | Lee J., Nishikawa R.M. | 2019 | Included in analysis |
| Detecting prostate cancer using deep learning convolution neural network with transfer learning approach | Cognitive Neurodynamics | Abbasi A.A., Hussain L., Awan I.A., Abbasi I., Majid A., Nadeem M.S.A., Chaudhary Q.-A. | 2020 | Included in analysis |
| Detection and characterization of MRI breast lesions using deep learning | Diagnostic and Interventional Imaging | Herent P., Schmauch B., Jehanno P., Dehaene O., Saillard C., Balleyguier C., Arfi-Rouche J., JÃ©gou S. | 2019 | Included in analysis |
| Detection and classification of the breast abnormalities in digital mammograms via regional Convolutional Neural Network. | Conference proceedings : | Al-Masni M.A., Al-Antari M.A., Park J.M., Gi G., Kim T.Y., Rivera P., Valarezo E., Han S.-M., Kim T.-S. | 2017 | Included in analysis |
| Detection of abnormalities in mammograms using deep features | Journal of Ambient Intelligence and Humanized Computing | Tavakoli N., Karimi M., Norouzi A., Karimi N., Samavi S., Soroushmehr S.M.R. | 2019 | Included in analysis |
| Detection of breast cancer via deep convolution neural networks using MRI images | Multimedia Tools and Applications | Yurttakal A.H., Erbay H., Ä°kizceli T., KaraÃ§avuÅŸ S. | 2020 | Included in analysis |
| Detection of lung cancer on chest CT images using minimum redundancy maximum relevance feature selection method with convolutional neural networks | Biocybernetics and Biomedical Engineering | ToÄŸaÃ§ar M., Ergen B., CÃ¶mert Z. | 2020 | Included in analysis |
| Detection of tumors on brain MRI images using the hybrid convolutional neural network architecture. | Medical Hypotheses | Cinar A., Yildirim M. | 2020 | Included in analysis |
| Determination of mammographic breast density using a deep convolutional neural network | British Journal of Radiology | Ciritsis A., Rossi C., De Martini I.V., Eberhard M., Marcon M., Becker A.S., Berger N., Boss A. | 2019 | Included in analysis |
| Diagnosis of Benign and Malignant Thyroid Nodules Using Combined Conventional Ultrasound and Ultrasound Elasticity Imaging. | IEEE Journal of Biomedical and Health Informatics | Qin P., Wu K., Hu Y., Zeng J., Chai X. | 2020 | Included in analysis |
| Diagnosis of focal liver lesions from ultrasound using deep learning. | Diagnostic and Interventional Imaging | Schmauch B., Herent P., Jehanno P., Dehaene O., Saillard C., Aube C., Luciani A., Lassau N., Jegou S. | 2019 | Included in analysis |
| Diagnosis of thyroid cancer using deep convolutional neural network models applied to sonographic images: a retrospective, multicohort, diagnostic study | The Lancet Oncology | Li X., Zhang S., Zhang Q., Wei X., Pan Y., Zhao J., Xin X., Qin C., Wang X., Li J., Yang F., Zhao Y., Yang M., Wang Q., Zheng Z., Zheng X., Yang X., Whitlow C.T., Gurcan M.N., Zhang L., Wang X., Pasche B.C., Gao M., Zhang W., Chen K. | 2019 | Included in analysis |
| Diagnosis of Thyroid Nodules: Performance of a Deep Learning Convolutional Neural Network Model vs. Radiologists. | Scientific reports | Park V.Y., Han K., Seong Y.K., Park M.H., Kim E.-K., Moon H.J., Yoon J.H., Kwak J.Y. | 2019 | Included in analysis |
| Diagnostic Efficiency of the Breast Ultrasound Computer-Aided Prediction Model Based on Convolutional Neural Network in Breast Cancer. | Journal of digital imaging | Zhang H., Han L., Chen K., Peng Y., Lin J. | 2020 | Included in analysis |
| Differential Diagnosis of Benign and Malignant Thyroid Nodules Using Deep Learning Radiomics of Thyroid Ultrasound Images. | European Journal of Radiology | Zhou H., Jin Y., Dai L., Zhang M., Qiu Y., wang K., Tian J., Zheng J. | 2020 | Included in analysis |
| Differentiation of Benign from Malignant Pulmonary Nodules by Using a Convolutional Neural Network to Determine Volume Change at Chest CT. | Radiology | Ohno Y., Aoyagi K., Yaguchi A., Seki S., Ueno Y., Kishida Y., Takenaka D., Yoshikawa T. | 2020 | Included in analysis |
| Differentiation of thyroid nodules on US using features learned and extracted from various convolutional neural networks. | Scientific reports | Lee E., Ha H., Kim H.J., Moon H.J., Byon J.H., Huh S., Son J., Yoon J., Han K., Kwak J.Y. | 2019 | Included in analysis |
| Digital mammographic tumor classification using transfer learning from deep convolutional neural networks | Journal of Medical Imaging | Huynh B.Q., Li H., Giger M.L. | 2016 | Included in analysis |
| Distinction between benign and malignant breast masses at breast ultrasound using deep learning method with convolutional neural network. | Japanese Journal of Radiology | Fujioka T., Kubota K., Mori M., Kikuchi Y., Katsuta L., Kasahara M., Oda G., Ishiba T., Nakagawa T., Tateishi U. | 2019 | Included in analysis |
| Distinction between phyllodes tumor and fibroadenoma in breast ultrasound using deep learning image analysis. | European Journal of Radiology Open | Stoffel E., Becker A.S., Wurnig M.C., Marcon M., Ghafoor S., Berger N., Boss A. | 2018 | Included in analysis |
| Effect of a deep learning framework-based computer-aided diagnosis system on the diagnostic performance of radiologists in differentiating between malignant and benign masses on breast ultrasonography. | Korean Journal of Radiology | Choi J.S., Han B.-K., Ko E.S., Bae J.M., Ko E.Y., Song S.H., Kwon M.-R., Shin J.H., Hahn S.Y. | 2019 | Included in analysis |
| Efficient anomaly detection with generative adversarial network for breast ultrasound imaging. | Diagnostics | Fujioka T., Kubota K., Mori M., Kikuchi Y., Katsuta L., Kimura M., Yamaga E., Adachi M., Oda G., Nakagawa T., Kitazume Y., Tateishi U. | 2020 | Included in analysis |
| Ensemble classification for predicting the malignancy level of pulmonary nodules on chest computed tomography images. | Oncology Letters | Xiao N., Qiang Y., Zia M.B., Wang S., Lian J. | 2020 | Included in analysis |
| Estimation of malignancy of pulmonary nodules at CT scans: Effect of computer-aided diagnosis on diagnostic performance of radiologists | Asia-Pacific Journal of Clinical Oncology | Liu J., Zhao L., Han X., Ji H., Liu L., He W. | 2020 | Included in analysis |
| Evaluate the Malignancy of Pulmonary Nodules Using the 3-D Deep Leaky Noisy-OR Network | IEEE Transactions on Neural Networks and Learning Systems | Liao F., Liang M., Li Z., Hu X., Song S. | 2019 | Included in analysis |
| Evaluating the performance of a deep learning-based computer-aided diagnosis (DL-CAD) system for detecting and characterizing lung nodules: Comparison with the performance of double reading by radiologists. | Thoracic Cancer | Li L., Liu Z., Huang H., Lin M., Luo D. | 2019 | Included in analysis |
| Evaluation of a deep learning-based computer-aided diagnosis system for distinguishing benign from malignant thyroid nodules in ultrasound images. | Medical Physics | Sun C., Zhang Y., Chang Q., Liu T., Zhang S., Wang X., Guo Q., Yao J., Sun W., Niu L. | 2020 | Included in analysis |
| Evaluation of deep learning detection and classification towards computer-aided diagnosis of breast lesions in digital X-ray mammograms | Computer Methods and Programs in Biomedicine | Al-antari M.A., Han S.-M., Kim T.-S. | 2020 | Included in analysis |
| Evolutionary image simplification for lung nodule classification with convolutional neural networks | International Journal of Computer Assisted Radiology and Surgery | LÃ¼ckehe D., von Voigt G. | 2018 | Included in analysis |
| Evolutionary pruning of transfer learned deep convolutional neural network for breast cancer diagnosis in digital breast tomosynthesis | Phys Med Biol | Samala RK, Chan HP, Hadjiiski LM, Helvie MA, Richter C, Cha K. | 2018 | Included in analysis |
| Explaining Deep Features Using Radiologist-Defined Semantic Features and Traditional Quantitative Features | Tomography (Ann Arbor, Mich.) | Paul R., Schabath M., Balagurunathan Y., Liu Y., Li Q., Gillies R., Hall L.O., Goldgof D.B. | 2019 | Included in analysis |
| Fusing learned representations from Riesz Filters and Deep CNN for lung tissue classification | Medical Image Analysis | Joyseeree R., OtÃ¡lora S., MÃ¼ller H., Depeursinge A. | 2019 | Included in analysis |
| GAN-based synthetic medical image augmentation for increased CNN performance in liver lesion classification. | Neurocomputing | Frid-Adar M., Diamant I., Klang E., Amitai M., Goldberger J., Greenspan H. | 2018 | Included in analysis |
| Generalization error analysis for deep convolutional neural network with transfer learning in breast cancer diagnosis. | Physics in medicine and biology | Samala R.K., Chan H.-P., Hadjiiski L.M., Helvie M.A., Richter C. | 2020 | Included in analysis |
| Highly accurate model for prediction of lung nodule malignancy with CT scans. | Scientific reports | Causey J.L., Zhang J., Ma S., Jiang B., Qualls J.A., Politte D.G., Prior F., Zhang S., Huang X. | 2018 | Included in analysis |
| Hybrid-feature-guided lung nodule type classification on CT images | Computers and Graphics (Pergamon) | Yuan J., Liu X., Hou F., Qin H., Hao A. | 2018 | Included in analysis |
| Identification of benign and malignant pulmonary nodules on chest CT using improved 3D U-Net deep learning framework. | European Journal of Radiology | Yang K., Liu J., Tang W., Zhang H., Zhang R., Gu J., Zhu R., Xiong J., Ru X., Wu J. | 2020 | Included in analysis |
| Identification of metastatic lymph nodes in MR imaging with faster region-based convolutional neural networks. | Cancer Research | Lu Y., Yu Q., Zhou Y., Liu G., Dong Q., Ma J., Ding L., Yao H., Zhang Z., Xiao G., An Q., Xi J., Yuan W., Lian Y., Zhang D., Zhao C., Yao Q., Liu W., Zhou X., Liu S., Wu Q., Xu W., Zhang J., Wang D., Sun Z., Gao Y., Zhang X., Hu J., Zhang M., Wang G., Zheng X., Wang L., Zhao J., Yang S. | 2018 | Included in analysis |
| Identifying pulmonary nodules or masses on chest radiography using deep learning: external validation and strategies to improve clinical practice. | Clinical Radiology | Liang C.-H., Liu Y.-C., Wu M.-T., Garcia-Castro F., Alberich-Bayarri A., Wu F.-Z. | 2020 | Included in analysis |
| Improving Accuracy of Lung Nodule Classification Using Deep Learning with Focal Loss. | Journal of Healthcare Engineering | Tran G.S., Nghiem T.P., Nguyen V.T., Luong C.M., Burie J.-C., Levin-Schwartz Y. | 2019 | Included in analysis |
| Improving Computer-Aided Detection Using Convolutional Neural Networks and Random View Aggregation | IEEE Transactions on Medical Imaging | Roth H.R., Lu L., Liu J., Yao J., Seff A., Cherry K., Kim L., Summers R.M. | 2016 | Included in analysis |
| International evaluation of an AI system for breast cancer screening | Nature | McKinney S.M., Sieniek M., Godbole V., Godwin J., Antropova N., Ashrafian H., Back T., Chesus M., Corrado G.C., Darzi A., Etemadi M., Garcia-Vicente F., Gilbert F.J., Halling-Brown M., Hassabis D., Jansen S., Karthikesalingam A., Kelly C.J., King D., Ledsam J.R., Melnick D., Mostofi H., Peng L., Reicher J.J., Romera-Paredes B., Sidebottom R., Suleyman M., Tse D., Young K.C., De Fauw J., Shetty S. | 2020 | Included in analysis |
| Joint Prostate Cancer Detection and Gleason Score Prediction in mp-MRI via FocalNet. | IEEE Transactions on Medical Imaging | Cao R., Mohammadian Bajgiran A., Afshari Mirak S., Shakeri S., Zhong X., Enzmann D., Raman S., Sung K. | 2019 | Included in analysis |
| Joint weakly and semi-supervised deep learning for localization and classification of masses in breast ultrasound images | IEEE Transactions on Medical Imaging | Shin S.Y., Lee S., Yun I.D., Kim S.M., Lee K.M. | 2019 | Included in analysis |
| Knowledge-based Collaborative Deep Learning for Benign-Malignant Lung Nodule Classification on Chest CT | IEEE Transactions on Medical Imaging | Xie Y., Xia Y., Zhang J., Song Y., Feng D., Fulham M., Cai W. | 2019 | Included in analysis |
| Large scale deep learning for computer aided detection of mammographic lesions | Medical Image Analysis | Kooi T., Litjens G., van Ginneken B., Gubern-MÃ©rida A., SÃ¡nchez C.I., Mann R., den Heeten A., Karssemeijer N. | 2017 | Included in analysis |
| Latent feature representation with depth directional long-term recurrent learning for breast masses in digital breast tomosynthesis | Physics in Medicine and Biology | Kim D.H., Kim S.T., Chang J.M., Ro Y.M. | 2017 | Included in analysis |
| Learning to detect chest radiographs containing pulmonary lesions using visual attention networks | Medical Image Analysis | Pesce E., Joseph Withey S., Ypsilantis P.-P., Bakewell R., Goh V., Montana G. | 2019 | Included in analysis |
| Local rotation invariance in 3D CNNs | Medical Image Analysis | Andrearczyk V., Fageot J., Oreiller V., Montet X., Depeursinge A. | 2020 | Included in analysis |
| Lung nodule classification using deep feature fusion in chest radiography. | Computerized Medical Imaging and Graphics | Wang C., Elazab A., Wu J., Hu Q. | 2017 | Included in analysis |
| Lung nodule classification using deep local global networks | International Journal of Computer Assisted Radiology and Surgery | Al-Shabi M., Lan B.L., Chan W.Y., Ng K.-H., Tan M. | 2019 | Included in analysis |
| Lung nodule detection and classification from Thorax CT-scan using RetinaNet with transfer learning | Journal of King Saud University - Computer and Information Sciences | Harsono I.W., Liawatimena S., Cenggoro T.W. | 2020 | Included in analysis |
| Lung nodules diagnosis based on evolutionary convolutional neural network | Multimedia Tools and Applications | da Silva G.L.F., da Silva Neto O.P., Silva A.C., de Paiva A.C., Gattass M. | 2017 | Included in analysis |
| Mass detection in digital breast tomosynthesis data using convolutional neural networks and multiple instance learning | Computers in Biology and Medicine | Yousefi M., KrzyÅ¼ak A., Suen C.Y. | 2018 | Included in analysis |
| Mediastinal lymph node malignancy detection in computed tomography images using fully convolutional network | Biocybernetics and Biomedical Engineering | Tekchandani H., Verma S., Londhe N.D. | 2020 | Included in analysis |
| MSCS-DeepLN: Evaluating lung nodule malignancy using multi-scale cost-sensitive neural networks | Medical Image Analysis | Xu X., Wang C., Guo J., Gan Y., Wang J., Bai H., Zhang L., Li W., Yi Z. | 2020 | Included in analysis |
| Multi-label transfer learning for the early diagnosis of breast cancer | Neurocomputing | Chougrad H., Zouaki H., Alheyane O. | 2020 | Included in analysis |
| Multilevel Contextual 3-D CNNs for False Positive Reduction in Pulmonary Nodule Detection. | IEEE Transactions on Biomedical Engineering | Dou Q., Chen H., Yu L., Qin J., Heng P.-A. | 2017 | Included in analysis |
| Multi-Level Cross Residual Network for Lung Nodule Classification. | Sensors (Basel, Switzerland) | Lyu J., Bi X., Ling S.H. | 2020 | Included in analysis |
| Multi-path convolutional neural network for lung cancer detection | Multidimensional Systems and Signal Processing | Sori W.J., Feng J., Liu S. | 2019 | Included in analysis |
| Multi-scale gradual integration CNN for false positive reduction in pulmonary nodule detection | Neural Networks | Kim B.-C., Yoon J.S., Choi J.-S., Suk H.-I. | 2019 | Included in analysis |
| Multiscale Mask R-CNN-Based Lung Tumor Detection Using PET Imaging. | Molecular Imaging | Zhang R., Cheng C., Zhao X., Li X. | 2019 | Included in analysis |
| Multi-View Mammographic Density Classification by Dilated and Attention-Guided Residual Learning. | IEEE/ACM transactions on computational biology and bioinformatics | Li C., Xu J., Liu Q., Zhou Y., Mou L., Pu Z., Xia Y., Zheng H., Wang S. | 2020 | Included in analysis |
| NODULe: Combining constrained multi-scale LoG filters with densely dilated 3D deep convolutional neural network for pulmonary nodule detection | Neurocomputing | Zhang J., Xia Y., Zeng H., Zhang Y. | 2018 | Included in analysis |
| Online Transfer Learning for Differential Diagnosis of Benign and Malignant Thyroid Nodules with Ultrasound Images. | IEEE transactions on bio-medical engineering | Zhou H., Wang K., Tian J. | 2020 | Included in analysis |
| Pattern classification for gastrointestinal stromal tumors by integration of radiomics and deep convolutional features. | IEEE Journal of Biomedical and Health Informatics | Ning Z., Luo J., Li Y., Han S., Feng Q., Xu Y., Chen W., Chen T., Zhang Y. | 2019 | Included in analysis |
| Perinodular and Intranodular Radiomic Features on Lung CT Images Distinguish Adenocarcinomas from Granulomas | Radiology | Beig N., Khorrami M., Alilou M., Prasanna P., Braman N., Orooji M., Rakshit S., Bera K., Rajiah P., Ginsberg J., Donatelli C., Thawani R., Yang M., Jacono F., Tiwari P., Velcheti V., Gilkeson R., Linden P., Madabhushi A. | 2019 | Included in analysis |
| Preliminary Study of Chronic Liver Classification on Ultrasound Images Using an Ensemble Model. | Ultrasonic imaging | Bharti P., Mittal D., Ananthasivan R. | 2018 | Included in analysis |
| Pulmonary Nodule Classification with Deep Convolutional Neural Networks on Computed Tomography Images. | Computational and Mathematical Methods in Medicine | Li W., Cao P., Zhao D., Wang J. | 2016 | Included in analysis |
| Pulmonary nodule classification with deep residual networks. | International Journal of Computer Assisted Radiology and Surgery | Nibali A., He Z., Wollersheim D. | 2017 | Included in analysis |
| Pulmonary Nodule Detection in CT Images: False Positive Reduction Using Multi-View Convolutional Networks | IEEE Transactions on Medical Imaging | Setio A.A.A., Ciompi F., Litjens G., Gerke P., Jacobs C., Van Riel S.J., Wille M.M.W., Naqibullah M., Sanchez C.I., Van Ginneken B. | 2016 | Included in analysis |
| Pulmonary nodule detection in CT scans with equivariant CNNs | Medical Image Analysis | Winkels M., Cohen T.S. | 2019 | Included in analysis |
| Reducing the number of unnecessary biopsies of US-BI-RADS 4a lesions through a deep learning method for residents-in-training: a cross-sectional study. | BMJ open | Zhao C., Xiao M., Liu H., Wang M., Wang H., Zhang J., Jiang Y., Zhu Q. | 2020 | Included in analysis |
| Searching for prostate cancer by fully automated magnetic resonance imaging classification: Deep learning versus non-deep learning | Scientific Reports | Wang X., Yang W., Weinreb J., Han J., Li Q., Kong X., Yan Y., Ke Z., Luo B., Liu T., Wang L. | 2017 | Included in analysis |
| Simultaneous detection and classification of breast masses in digital mammograms via a deep learning YOLO-based CAD system. | Computer Methods & Programs in Biomedicine | Al-Masni MA, Al-Antari MA, Park JM, Gi G, Kim TY, Rivera P, Valarezo E, Choi MT, Han SM, Kim TS | 2018 | Included in analysis |
| Single-view 2D CNNs with fully automatic non-nodule categorization for false positive reduction in pulmonary nodule detection | Computer Methods and Programs in Biomedicine | Eun H., Kim D., Jung C., Kim C. | 2018 | Included in analysis |
| Thyroid Nodule Classification in Ultrasound Images by Fine-Tuning Deep Convolutional Neural Network. | Journal of digital imaging | Chi J., Walia E., Babyn P., Wang J., Groot G., Eramian M. | 2017 | Included in analysis |
| Toward an Expert Level of Lung Cancer Detection and Classification Using a Deep Convolutional Neural Network. | Oncologist | Zhang C., Sun X., Dang K., Li K., Guo X.-W., Chang J., Yu Z.-Q., Huang F.-Y., Wu Y.-S., Liang Z., Liu Z.-Y., Zhang X.-G., Gao X.-L., Huang S.-H., Qin J., Feng W.-N., Zhou T., Zhang Y.-B., Fang W.-J., Zhao M.-F., Yang X.-N., Zhou Q., Wu Y.-L., Zhong W.-Z. | 2019 | Included in analysis |
| Toward classifying small lung nodules with hyperparameter optimization of convolutional neural networks | Computational Intelligence | Lima L.L., Ferreira Junior J.R., Oliveira M.C. | 2020 | Included in analysis |
| Transfer Learning From Convolutional Neural Networks for Computer-Aided Diagnosis: A Comparison of Digital Breast Tomosynthesis and Full-Field Digital Mammography. | Academic Radiology | Mendel K, Li H, Sheth D, Giger M | 2019 | Included in analysis |
| Tumor detection in automated breast ultrasound using 3-D CNN and prioritized candidate aggregation | IEEE Transactions on Medical Imaging | Chiang T.-C., Huang Y.-S., Chen R.-T., Huang C.-S., Chang R.-F. | 2019 | Included in analysis |
| Ultrasonographic Thyroid Nodule Classification Using a Deep Convolutional Neural Network with Surgical Pathology. | Journal of digital imaging | Kwon S.W., Choi I.J., Kang J.Y., Jang W.I., Lee G.-H., Lee M.-C. | 2020 | Included in analysis |
| Ultrasound image analysis using deep learning algorithm for the diagnosis of thyroid nodules. | Medicine | Song J., Chai Y.J., Masuoka H., Park S.-W., Kim S.-J., Choi J.Y., Kong H.-J., Lee K.E., Lee J., Kwak N., Yi K.H., Miyauchi A. | 2019 | Included in analysis |
| Usefulness of deep learning analysis for the diagnosis of malignancy in intraductal papillary mucinous neoplasms of the pancreas. | Clinical and Translational Gastroenterology | Kuwahara T., Hara K., Mizuno N., Okuno N., Matsumoto S., Obata M., Kurita Y., Koda H., Toriyama K., Onishi S., Ishihara M., Tanaka T., Tajika M., Niwa Y. | 2019 | Included in analysis |
| Using Deep Learning for Classification of Lung Nodules on Computed Tomography Images. | Journal of Healthcare Engineering | Song Q.Z., Zhao L., Luo X.K., Dou X.C. | 2017 | Included in analysis |
| Using Multi-level Convolutional Neural Network for Classification of Lung Nodules on CT images. | Conference proceedings : | Lyu J., Ling S.H. | 2018 | Included in analysis |
| Validation, comparison, and combination of algorithms for automatic detection of pulmonary nodules in computed tomography images: The LUNA16 challenge | Medical Image Analysis | Setio A.A.A., Traverso A., de Bel T., Berens M.S.N., Bogaard C.V.D., Cerello P., Chen H., Dou Q., Fantacci M.E., Geurts B., Gugten R.V.D., Heng P.A., Jansen B., de Kaste M.M.J., Kotov V., Lin J.Y.-H., Manders J.T.M.C., SÃ³Ã±ora-Mengana A., GarcÃ­a-Naranjo J.C., Papavasileiou E., Prokop M., Saletta M., Schaefer-Prokop C.M., Scholten E.T., Scholten L., Snoeren M.M., Torres E.L., Vandemeulebroucke J., Walasek N., Zuidhof G.C.A., Ginneken B.V., Jacobs C. | 2017 | Included in analysis |
| Visually interpretable deep network for diagnosis of breast masses on mammograms. | Physics in Medicine & Biology | Kim ST, Lee JH, Lee H, Ro YM | 2018 | Included in analysis |
| Automatic classification of tissue malignancy for breast carcinoma diagnosis | Computers in Biology and Medicine | FondÃ³n I., Sarmiento A., GarcÃ­a A.I., Silvestre M., Eloy C., PolÃ³nia A., Aguiar P. | 2018 | Model receives inputs other than radiological image |
| Deep Learning Role in Early Diagnosis of Prostate Cancer. | Technology in Cancer Research & Treatment | Reda I, Khalil A, Elmogy M, Abou El-Fetouh A, Shalaby A, Abou El-Ghar M, Elmaghraby A, Ghazal M, El-Baz A | 2018 | Model receives inputs other than radiological image |
| Extraction of BI-RADS findings from breast ultrasound reports in Chinese using deep learning approaches | International Journal of Medical Informatics | Miao S., Xu T., Wu Y., Xie H., Wang J., Jing S., Zhang Y., Zhang X., Yang Y., Zhang X., Shan T., Wang L., Xu H., Wang S., Liu Y. | 2018 | Model receives inputs other than radiological image |
| Knowledge-guided synthetic medical image adversarial augmentation for ultrasonography thyroid nodule classification. | Computer Methods and Programs in Biomedicine | Shi G., Wang J., Qiang Y., Yang X., Zhao J., Hao R., Yang W., Du Q., Kazihise N.G.-F. | 2020 | Model receives inputs other than radiological image |
| Solitary solid pulmonary nodules: a CT-based deep learning nomogram helps differentiate tuberculosis granulomas from lung adenocarcinomas. | European Radiology | Feng B., Chen X.M., Chen Y.H., Lu S.L., Liu K.F., Li K.W., Liu Z.S., Hao Y.X., Li Z., Zhu Z.B., Yao N., Liang G.Y., Zhang J.Y., Long W.S., Liu X.G. | 2020 | Model receives inputs other than radiological image |
| A collaborative computer aided diagnosis (C-CAD) system with eye-tracking, sparse attentional model, and deep learning. | Medical Image Analysis | Khosravan N, Celik H, Turkbey B, Jones EC, Wood B, Bagci U | 2018 | No CNN model |
| A comprehensive non-invasive framework for diagnosing prostate cancer. | Computers in Biology and Medicine | Reda I., Shalaby A., Elmogy M., Elfotouh A.A., Khalifa F., El-Ghar M.A., Hosseini-Asl E., Gimel'farb G., Werghi N., El-Baz A. | 2017 | No CNN model |
| A generalized deep learning-based diagnostic system for early diagnosis of various types of pulmonary nodules. | Technology in Cancer Research and Treatment | Shaffie A., Soliman A., Fraiwan L., Ghazal M., Taher F., Dunlap N., Wang B., van Berkel V., Keynton R., Elmaghraby A., El-Baz A. | 2018 | No CNN model |
| Application of fast curvelet Tsallis entropy and kernel random vector functional link network for automated detection of multiclass brain abnormalities | Computerized Medical Imaging and Graphics | Nayak D.R., Dash R., Majhi B., Acharya U.R. | 2019 | No CNN model |
| CT Image-based Decision Support System for Categorization of Liver Metastases Into Primary Cancer Sites: Initial Results | Academic Radiology | Ben-Cohen A., Klang E., Diamant I., Rozendorn N., Raskin S.P., Konen E., Amitai M.M., Greenspan H. | 2017 | No CNN model |
| Decision Support System for Lung Cancer Using PET/CT and Microscopic Images. | Advances in Experimental Medicine and Biology | Teramoto A., Yamada A., Tsukamoto T., Imaizumi K., Toyama H., Saito K., Fujita H. | 2020 | No CNN model |
| Deep learning can be used to train naive, nonprofessional observers to detect diagnostic visual patterns of certain cancers in mammograms: A proof-of-principle study. | Journal of Medical Imaging | Hegde J. | 2020 | No CNN model |
| Deep learning in mammography diagnostic accuracy of a multipurpose image analysis software in the detection of breast cancer. | Investigative Radiology | Becker A.S., Marcon M., Ghafoor S., Wurnig M.C., Frauenfelder T., Boss A. | 2017 | No CNN model |
| Deep learning-based radiomic features for improving neoadjuvant chemoradiation response prediction in locally advanced rectal cancer. | Physics in medicine and biology | Fu J., Zhong X., Li N., van Dams R., Lewis J.H., Sung K., Raldow A.C., Jin J., Qi S.X. | 2020 | No CNN model |
| Deep lessons learned: Radiology, oncology, pathology, and computer science experts unite around artificial intelligence to strive for earlier pancreatic cancer diagnosis | Diagnostic and Interventional Imaging | Weisberg E.M., Chu L.C., Park S., Yuille A.L., Kinzler K.W., Vogelstein B., Fishman E.K. | 2020 | No CNN model |
| DeepCAD: A computer-aided diagnosis system for mammographic masses using deep invariant features | Computers | Abbas Q. | 2016 | No CNN model |
| Detection of prostate cancer using temporal sequences of ultrasound data: a large clinical feasibility study | International Journal of Computer Assisted Radiology and Surgery | Azizi S., Imani F., Ghavidel S., Tahmasebi A., Kwak J.T., Xu S., Turkbey B., Choyke P., Pinto P., Wood B., Mousavi P., Abolmaesumi P. | 2016 | No CNN model |
| Diagnostic Value of Breast Lesions Between Deep Learning-Based Computer-Aided Diagnosis System and Experienced Radiologists: Comparison the Performance Between Symptomatic and Asymptomatic Patients. | Frontiers in Oncology | Xiao M., Zhao C., Li J., Zhang J., Liu H., Wang M., Ouyang Y., Zhang Y., Jiang Y., Zhu Q. | 2020 | No CNN model |
| Medical Sign Recognition of Lung Nodules Based on Image Retrieval with Semantic Features and Supervised Hashing | Journal of Computer Science and Technology | Zhao J.-J., Pan L., Zhao P.-F., Tang X.-X. | 2017 | No CNN model |
| Multimodal feature learning and fusion on B-mode ultrasonography and sonoelastography using point-wise gated deep networks for prostate cancer diagnosis | Biomedizinische Technik | Zhang Q., Xiong J., Cai Y., Shi J., Xu S., Zhang B. | 2019 | No CNN model |
| Optimal deep learning model for classification of lung cancer on CT images | Future Generation Computer Systems | Lakshmanaprabu S.K., Mohanty S.N., Shankar K., Arunkumar N., Ramirez G. | 2019 | No CNN model |
| Quantitative vessel tortuosity: A potential CT imaging biomarker for distinguishing lung granulomas from adenocarcinomas | Scientific Reports | Alilou M., Orooji M., Beig N., Prasanna P., Rajiah P., Donatelli C., Velcheti V., Rakshit S., Yang M., Jacono F., Gilkeson R., Linden P., Madabhushi A. | 2018 | No CNN model |
| Stacked deep polynomial network based representation learning for tumor classification with small ultrasound image dataset. | Neurocomputing | Shi J., Zhou S., Liu X., Zhang Q., Lu M., Wang T. | 2016 | No CNN model |
| Subclass based parallel learning neural network for classification of masses in mammograms | Design Automation for Embedded Systems | Sivakrithika V., Dinakaran K. | 2018 | No CNN model |
| Tumor net (T-net) for classification of multi tumors in brain MR images | International Journal of Advanced Trends in Computer Science and Engineering | Anil Kumar B., Rajesh Kumar P. | 2020 | No CNN model |
| Ultrasound computer-aided diagnosis (CAD) based on the thyroid imaging reporting and Data System (TI-RADS) to distinguish benign from malignant thyroid nodules and the diagnostic performance of radiologists with different diagnostic experience. | Medical Science Monitor | Jin Z., Zhu Y., Zhang S., Xie F., Zhang M., Zhang Y., Tian X., Zhang J., Luo Y., Cao J. | 2020 | No CNN model |
| Ultrasound-Based Diagnosis of Breast Tumor with Parameter Transfer Multilayer Kernel Extreme Learning Machine. | Conference proceedings : | Fei X., Zhou W., Shen L., Chang C., Zhou S., Shi J. | 2019 | No CNN model |
| Can we reduce the workload of mammographic screening by automatic identification of normal exams with artificial intelligence? A feasibility study | European Radiology | Rodriguez-Ruiz A., LÃ¥ng K., Gubern-Merida A., Teuwen J., Broeders M., Gennaro G., Clauser P., Helbich T.H., Chevalier M., Mertelmeier T., Wallis M.G., Andersson I., Zackrisson S., Sechopoulos I., Mann R.M. | 2019 | Non-diagnostic task |
| Clinical evaluation of atlas and deep learning based automatic contouring for lung cancer | Radiotherapy and Oncology | Lustberg T., van Soest J., Gooding M., Peressutti D., Aljabar P., van der Stoep J., van Elmpt W., Dekker A. | 2018 | Non-diagnostic task |
| Comparison of a deep learning risk score and standard mammographic density score for breast cancer risk prediction. | Radiology | Dembrower K., Liu Y., Azizpour H., Eklund M., Smith K., Lindholm P., Strand F. | 2020 | Non-diagnostic task |
| DC-AL GAN: Pseudoprogression and true tumor progression of glioblastoma multiform image classification based on DCGAN and AlexNet. | Medical Physics | Li M., Tang H., Chan M.D., Zhou X., Qian X. | 2020 | Non-diagnostic task |
| Discrimination between transient and persistent subsolid pulmonary nodules on baseline CT using deep transfer learning. | European Radiology | Huang C., Lv W., Zhou C., Mao L., Xu Q., Qi L., Xia F., Li X., Zhang Q., Zhang L., Lu G. | 2020 | Non-diagnostic task |
| Prediction of lymph node maximum standardized uptake value in patients with cancer using a 3D convolutional neural network: A proof-of-concept study | American Journal of Roentgenology | Shaish H., Mutasa S., Makkar J., Chang P., Schwartz L., Ahmed F. | 2019 | Non-diagnostic task |
| Prostate cancer detection from multi-institution multiparametric MRIs using deep convolutional neural networks. | Journal of Medical Imaging | Sumathipala Y., Lay N., Turkbey B., Smith C., Choyke P.L., Summers R.M. | 2018 | Non-diagnostic task |
| RAMS: Remote and automatic mammogram screening | Computers in Biology and Medicine | Cogan T., Cogan M., Tamil L. | 2019 | Non-diagnostic task |
| ABCD rule and pre-trained CNNs for melanoma diagnosis | Multimedia Tools and Applications | Moura N., Veras R., Aires K., Machado V., Silva R., AraÃºjo F., Claro M. | 2019 | Non-radiological task |
| Analysis on the potential of an EAâ€“surrogate modelling tandem for deep learning parametrization: an example for cancer classification from medical images | Neural Computing and Applications | Stoean R. | 2020 | Non-radiological task |
| Automated assessment of breast cancer margin in optical coherence tomography images via pretrained convolutional neural network. | Journal of biophotonics | Singla N., Dubey K., Srivastava V. | 2019 | Non-radiological task |
| Breast Cancer Multi-classification through Deep Neural Network and Hierarchical Classification Approach | Multimedia Tools and Applications | Murtaza G., Shuib L., Mujtaba G., Raza G. | 2020 | Non-radiological task |
| Classification of breast cancer histology images using incremental boosting convolution networks | Information Sciences | Vo D.M., Nguyen N.-Q., Lee S.-W. | 2019 | Non-radiological task |
| Computer aided detection of prostate cancer using multiwavelength photoacoustic data with convolutional neural network. | Biomedical Signal Processing and Control | Dhengre N., Sinha S., Chinni B., Dogra V., Rao N. | 2020 | Non-radiological task |
| Computer-aided diagnosis of endobronchial ultrasound images using convolutional neural network. | Computer Methods and Programs in Biomedicine | Chen C.-H., Lee Y.-W., Huang Y.-S., Lan W.-R., Chang R.-F., Tu C.-Y., Chen C.-Y., Liao W.-C. | 2019 | Non-radiological task |
| Computer-assisted medical image classification for early diagnosis of oral cancer employing deep learning algorithm. | Journal of Cancer Research and Clinical Oncology | Jeyaraj P.R., Samuel Nadar E.R. | 2019 | Non-radiological task |
| Deep Model with Siamese Network for Viable and Necrotic Tumor Regions Assessment in Osteosarcoma. | Medical physics | Fu Y., Xue P., Ji H., Cui W., Dong E. | 2020 | Non-radiological task |
| Detection of cervical cancer cells based on strong feature CNN-SVM network | Neurocomputing | Dongyao Jia A., Zhengyi Li B., Chuanwang Zhang C. | 2020 | Non-radiological task |
| Diagnostic ability of artificial intelligence using deep learning analysis of cyst fluid in differentiating malignant from benign pancreatic cystic lesions | Scientific Reports | Kurita Y., Kuwahara T., Hara K., Mizuno N., Okuno N., Matsumoto S., Obata M., Koda H., Tajika M., Shimizu Y., Nakajima A., Kubota K., Niwa Y. | 2019 | Non-radiological task |
| EMS-Net: Ensemble of Multiscale Convolutional Neural Networks for Classification of Breast Cancer Histology Images | Neurocomputing | Yang Z., Ran L., Zhang S., Xia Y., Zhang Y. | 2019 | Non-radiological task |
| Ensembled deep convolution neural network-based breast cancer classification with misclassification reduction algorithms | Multimedia Tools and Applications | Murtaza G., Shuib L., Wahab A.W.A., Mujtaba G., Raza G. | 2020 | Non-radiological task |
| Extracting subset of relevant features for breast cancer to improve accuracy of classifier | International Journal of Innovative Technology and Exploring Engineering | Saturi R., Dara R., Prem Chand P. | 2019 | Non-radiological task |
| Fast ScanNet: Fast and Dense Analysis of Multi-Gigapixel Whole-Slide Images for Cancer Metastasis Detection. | IEEE Transactions on Medical Imaging | Lin H., Chen H., Graham S., Dou Q., Rajpoot N., Heng P.-A. | 2019 | Non-radiological task |
| Guided Soft Attention Network for Classification of Breast Cancer Histopathology Images | IEEE Transactions on Medical Imaging | Yang H., Kim J.-Y., Kim H., Adhikari S.P. | 2020 | Non-radiological task |
| Hybrid Transfer Learning for Classification of Uterine Cervix Images for Cervical Cancer Screening | Journal of Digital Imaging | Kudva V., Prasad K., Guruvare S. | 2020 | Non-radiological task |
| MuDeRN: Multi-category classification of breast histopathological image using deep residual networks | Artificial Intelligence in Medicine | Gandomkar Z., Brennan P.C., Mello-Thoms C. | 2018 | Non-radiological task |
| Multi-class breast cancer classification using deep learning convolutional neural network | International Journal of Advanced Computer Science and Applications | Nawaz M., Sewissy A.A., Soliman T.A. | 2018 | Non-radiological task |
| Optimize Transfer Learning for Lung Diseases in Bronchoscopy Using a New Concept: Sequential Fine-Tuning. | IEEE Journal of Translational Engineering in Health and Medicine | Tan T., Li Z., Liu H., Zanjani F.G., Ouyang Q., Tang Y., Hu Z., Li Q. | 2018 | Non-radiological task |
| Using deep convolutional neural networks to identify and classify tumor-associated stroma in diagnostic breast biopsies. | Modern Pathology | Ehteshami Bejnordi B., Mullooly M., Pfeiffer R.M., Fan S., Vacek P.M., Weaver D.L., Herschorn S., Brinton L.A., van Ginneken B., Karssemeijer N., Beck A.H., Gierach G.L., van der Laak J.A.W.M., Sherman M.E. | 2018 | Non-radiological task |
| Computer-aided detection of brain tumor from magnetic resonance images using deep learning network | Journal of Ambient Intelligence and Humanized Computing | Chanu M.M., Thongam K. | 2020 | Not available in English language |
| Application of deep transfer learning for automated brain abnormality classification using MR images. | Cognitive Systems Research | Talo M., Baloglu U.B., Yildirim O., Rajendra Acharya U. | 2019 | Task other than cancer diagnosis |
| Automated detection of focal cortical dysplasia using a deep convolutional neural network | Computerized Medical Imaging and Graphics | Wang H., Ahmed S.N., Mandal M. | 2020 | Task other than cancer diagnosis |
| Automated detection of vulnerable plaque in intravascular ultrasound images. | Medical and Biological Engineering and Computing | Jun T.J., Kang S.-J., Lee J.-G., Kweon J., Na W., Kang D., Kim D., Kim Y.-H. | 2019 | Task other than cancer diagnosis |
| Automatic quantification of calcifications in the coronary arteries and thoracic aorta on radiotherapy planning CT scans of Western and Asian breast cancer patients | Radiotherapy and Oncology | Gernaat S.A.M., van Velzen S.G.M., Koh V., Emaus M.J., IÅ¡gum I., Lessmann N., Moes S., Jacobson A., Tan P.W., Grobbee D.E., van den Bongard D.H.J., Tang J.I., Verkooijen H.M. | 2018 | Task other than cancer diagnosis |
| Auxiliary diagnosis of developmental dysplasia of the hip by automated detection of Sharp's angle on standardized anteroposterior pelvic radiographs. | Medicine (United States) | Li Q., Zhong L., Huang H., Qin Y., Zhou Z., Liu H., Yang W., Qin M., Wang Y., Zhou T., Wang D., Wang J., Xu M., Huang Y. | 2019 | Task other than cancer diagnosis |
| Cardiac sarcoidosis classification with deep convolutional neural network-based features using polar maps | Computers in Biology and Medicine | Togo R., Hirata K., Manabe O., Ohira H., Tsujino I., Magota K., Ogawa T., Haseyama M., Shiga T. | 2019 | Task other than cancer diagnosis |
| Classification of Carotid Artery Intima Media Thickness Ultrasound Images with Deep Learning | Journal of Medical Systems | SavaÅŸ S., TopaloÄŸlu N., KazcÄ± Ã–., KoÅŸar P.N. | 2019 | Task other than cancer diagnosis |
| Classification of CT brain images based on deep learning networks. | Computer Methods and Programs in Biomedicine | Gao X.W., Hui R., Tian Z. | 2017 | Task other than cancer diagnosis |
| Computer-aided detection and diagnosis of microcalcification clusters on full field digital mammograms based on deep learning method using neutrosophic boosting | Multimedia Tools and Applications | Cai G., Guo Y., Chen W., Zeng H., Zhou Y., Lu Y. | 2020 | Task other than cancer diagnosis |
| Deep learning, computer-aided radiography reading for tuberculosis: a diagnostic accuracy study from a tertiary hospital in India. | Scientific reports | Nash M., Kadavigere R., Andrade J., Sukumar C.A., Chawla K., Shenoy V.P., Pande T., Huddart S., Pai M., Saravu K. | 2020 | Task other than cancer diagnosis |
| Deep learning-based detection system for multiclass lesions on chest radiographs: comparison with observer readings. | European Radiology | Park S., Lee S.M., Lee K.H., Jung K.-H., Bae W., Choe J., Seo J.B. | 2020 | Task other than cancer diagnosis |
| Detection of gastritis by a deep convolutional neural network from double-contrast upper gastrointestinal barium X-ray radiography | Journal of Gastroenterology | Togo R., Yamamichi N., Mabe K., Takahashi Y., Takeuchi C., Kato M., Sakamoto N., Ishihara K., Ogawa T., Haseyama M. | 2019 | Task other than cancer diagnosis |
| Development and Validation of a Deep Learning-Based Automated Detection Algorithm for Major Thoracic Diseases on Chest Radiographs. | JAMA network open | Hwang E.J., Park S., Jin K.-N., Kim J.I., Choi S.Y., Lee J.H., Goo J.M., Aum J., Yim J.-J., Cohen J.G., Ferretti G.R., Park C.M. | 2019 | Task other than cancer diagnosis |
| Ensemble deep learning for tuberculosis detection using chest X-ray and canny edge detected images | IAES International Journal of Artificial Intelligence | Hwa S.K.T., Hijazi M.H.A., Bade A., Yaakob R., Jeffree M.S. | 2019 | Task other than cancer diagnosis |
| Evaluation of a computer-aided method for measuring the Cobb angle on chest X-rays. | European Spine Journal | Pan Y., Chen Q., Chen T., Wang H., Zhu X., Fang Z., Lu Y. | 2019 | Task other than cancer diagnosis |
| Gender imbalance in medical imaging datasets produces biased classifiers for computer-aided diagnosis | Proceedings of the National Academy of Sciences of the United States of America | Larrazabal A.J., Nieto N., Peterson V., Milone D.H., Ferrante E. | 2020 | Task other than cancer diagnosis |
| Hybrid deep learning for detecting lung diseases from X-ray images | Informatics in Medicine Unlocked | Bharati S., Podder P., Mondal M.R.H. | 2020 | Task other than cancer diagnosis |
| Hybrid resampling and multi-feature fusion for automatic recognition of cavity imaging sign in lung CT | Future Generation Computer Systems | Han G., Liu X., Zhang H., Zheng G., Soomro N.Q., Wang M., Liu W. | 2019 | Task other than cancer diagnosis |
| Improving multi-label chest X-ray disease diagnosis by exploiting disease and health labels dependencies | Multimedia Tools and Applications | Ge Z., Mahapatra D., Chang X., Chen Z., Chi L., Lu H. | 2020 | Task other than cancer diagnosis |
| Learning-based vertebra localization and labeling in 3D CT data of possibly incomplete and pathological spines. | Computer Methods and Programs in Biomedicine | Jakubicek R., Chmelik J., Jan J., Ourednicek P., Lambert L., Gavelli G. | 2020 | Task other than cancer diagnosis |
| Multiple instance learning for histopathological breast cancer image classification | Expert Systems with Applications | Sudharshan P.J., Petitjean C., Spanhol F., Oliveira L.E., Heutte L., Honeine P. | 2019 | Task other than cancer diagnosis |
| Optimal Deep Neural Network model based multimodality fused medical image classification | Physical Communication | Subbiah Parvathy V., Pothiraj S., Sampson J. | 2020 | Task other than cancer diagnosis |
| Performance of Qure.ai automatic classifiers against a large annotated database of patients with diverse forms of tuberculosis. | PLoS ONE | Engle E., Gabrielian A., Long A., Hurt D.E., Rosenthal A. | 2020 | Task other than cancer diagnosis |
| Pneumonia Detection in Chest X-Ray Dose-Equivalent CT: Impact of Dose Reduction on Detectability by Artificial Intelligence. | Academic Radiology | Schwyzer M., Martini K., Skawran S., Messerli M., Frauenfelder T. | 2020 | Task other than cancer diagnosis |
| Towards large-scale case-finding: training and validation of residual networks for detection of chronic obstructive pulmonary disease using low-dose CT.  **Supplementary Table 2. References for articles included in abstract/full-text screen. Reasons for exclusion are provided where applicable.**   \| **Item** \| **Compliant Studies** (N) \| **Applicable Studies** (N) \| **Compliance** (%) \| **95% CI** (%) \| \| --- \| --- \| --- \| --- \| --- \| \| 1 \| 170 \| 186 \| 91% \| 86-95% \| \| 2 \| 131 \| 186 \| 70% \| 63-77% \| \| 3 \| 183 \| 186 \| 98% \| 95-100% \| \| 4a \| 171 \| 186 \| 92% \| 87-95% \| \| 4b \| 9 \| 186 \| 5% \| 2-9% \| \| 5 \| 62 \| 186 \| 33% \| 27-41% \| \| 6 \| 47 \| 186 \| 25% \| 19-32% \| \| 7a \| 174 \| 186 \| 94% \| 89-97% \| \| 7b \| 135 \| 186 \| 73% \| 66-79% \| \| 7c \| 94 \| 186 \| 51% \| 43-58% \| \| 7d \| 69 \| 186 \| 37% \| 30-44% \| \| 7e \| 89 \| 186 \| 48% \| 40-55% \| \| 7f \| 32 \| 186 \| 17% \| 12-23% \| \| 8 \| 51 \| 186 \| 27% \| 21-34% \| \| 9 \| 104 \| 186 \| 56% \| 48-63% \| \| 10 \| 74 \| 186 \| 40% \| 33-47% \| \| 11 \| 186 \| 186 \| 100% \| 98-100% \| \| 12 \| 3 \| 186 \| 2% \| 0-5% \| \| 13 \| 31 \| 186 \| 17% \| 12-23% \| \| 14a \| 1 \| 186 \| 1% \| 0-3% \| \| 14b \| 45 \| 186 \| 24% \| 18-31% \| \| 15a \| 5 \| 186 \| 3% \| 1-6% \| \| 15b \| 93 \| 186 \| 50% \| 43-57% \| \| 16a \| 75 \| 186 \| 40% \| 33-48% \| \| 16b \| 0 \| 98 \| 0% \| 0-4% \| \| 17 \| 22 \| 186 \| 12% \| 8-17% \| \| 18 \| 30 \| 186 \| 16% \| 11-22% \| \| 19a \| 161 \| 186 \| 87% \| 81-91% \| \| 19b \| 1 \| 186 \| 1% \| 0-3% \| \| 19c \| 133 \| 186 \| 72% \| 64-78% \| \| 20 \| 136 \| 179 \| 76% \| 69-82% \| \| 21 \| 58 \| 179 \| 32% \| 26-40% \| \| 22a \| 119 \| 179 \| 66% \| 59-73% \| \| 22b \| 35 \| 179 \| 20% \| 14-26% \| \| 23 \| 74 \| 179 \| 41% \| 34-49% \| \| 24 \| 122 \| 179 \| 68% \| 61-75% \| \| 25a \| 98 \| 179 \| 55% \| 47-62% \| \| 25b \| 78 \| 179 \| 44% \| 36-51% \| \| 25c \| 109 \| 179 \| 61% \| 53-68% \| \| 25d \| 110 \| 179 \| 61% \| 54-69% \| \| 25e \| 98 \| 179 \| 55% \| 47-62% \| \| 25f \| 93 \| 179 \| 52% \| 44-59% \| \| 26 \| 123 \| 179 \| 69% \| 61-75% \| \| 27 \| 2 \| 29 \| 7% \| 1-23% \| \| 28 \| 67 \| 186 \| 36% \| 29-43% \| \| 29 \| 72 \| 186 \| 39% \| 32-46% \| \| 30 \| 22 \| 186 \| 12% \| 8-17% \| \| 31 \| 17 \| 186 \| 9% \| 5-14% \| \| 32 \| 28 \| 186 \| 15% \| 10-21% \| \| 33 \| 14 \| 186 \| 8% \| 4-12% \| \| 34 \| 18 \| 186 \| 10% \| 6-15% \| \| 35a \| 91 \| 186 \| 49% \| 42-56% \| \| 35b \| 31 \| 186 \| 17% \| 12-23% \| \| 35c \| 77 \| 186 \| 41% \| 34-49% \| \| 36 \| 75 \| 186 \| 40% \| 33-48% \| \| 37 \| 44 \| 186 \| 24% \| 18-30% \| \| 38 \| 85 \| 186 \| 46% \| 38-53% \| \| 39 \| 69 \| 186 \| 37% \| 30-44% \| \| 40 \| 13 \| 186 \| 7% \| 4-12% \| \| 41 \| 2 \| 186 \| 1% \| 0-4% \| \| 42 \| 18 \| 186 \| 10% \| 6-15% \| | The Lancet Digital Health | Tang L.Y.W., Coxson H.O., Lam S., Leipsic J., Tam R.C., Sin D.D. | 2020 | Task other than cancer diagnosis |

**Supplementary Table 3. Compliance for each item. Some items were not applicable to all studies. Compliance was measured in the applicable subset (exemptions are provided in Table 1).**

| **Item** | **Compliant Studies** (N) | **Applicable Studies** (N) | **Compliance** (%) | **95% CI** (%) |
| --- | --- | --- | --- | --- |
| 1 | 170 | 186 | 91% | 86-95% |
| 2 | 131 | 186 | 70% | 63-77% |
| 3 | 183 | 186 | 98% | 95-100% |
| 4a | 171 | 186 | 92% | 87-95% |
| 4b | 9 | 186 | 5% | 2-9% |
| 5 | 62 | 186 | 33% | 27-41% |
| 6 | 47 | 186 | 25% | 19-32% |
| 7a | 174 | 186 | 94% | 89-97% |
| 7b | 135 | 186 | 73% | 66-79% |
| 7c | 94 | 186 | 51% | 43-58% |
| 7d | 69 | 186 | 37% | 30-44% |
| 7e | 89 | 186 | 48% | 40-55% |
| 7f | 32 | 186 | 17% | 12-23% |
| 8 | 51 | 186 | 27% | 21-34% |
| 9 | 104 | 186 | 56% | 48-63% |
| 10 | 74 | 186 | 40% | 33-47% |
| 11 | 186 | 186 | 100% | 98-100% |
| 12 | 3 | 186 | 2% | 0-5% |
| 13 | 31 | 186 | 17% | 12-23% |
| 14 | 46 | 186 | 25% | 19-32% |
| 15a | 5 | 186 | 3% | 1-6% |
| 15b | 93 | 186 | 50% | 43-57% |
| 16a | 75 | 186 | 40% | 33-48% |
| 16b | 7 | 88 | 8% | 3-16% |
| 17 | 22 | 186 | 12% | 8-17% |
| 18 | 30 | 186 | 16% | 11-22% |
| 19a | 161 | 186 | 87% | 81-91% |
| 19b | 1 | 186 | 1% | 0-3% |
| 19c | 133 | 186 | 72% | 64-78% |
| 20 | 136 | 179 | 76% | 69-82% |
| 21 | 58 | 179 | 32% | 26-40% |
| 22a | 119 | 179 | 66% | 59-73% |
| 22b | 35 | 179 | 20% | 14-26% |
| 23 | 74 | 179 | 41% | 34-49% |
| 24 | 122 | 179 | 68% | 61-75% |
| 25a | 98 | 179 | 55% | 47-62% |
| 25b | 78 | 179 | 44% | 36-51% |
| 25c | 109 | 179 | 61% | 53-68% |
| 25d | 110 | 179 | 61% | 54-69% |
| 25e | 98 | 179 | 55% | 47-62% |
| 25f | 93 | 179 | 52% | 44-59% |
| 26 | 123 | 179 | 69% | 61-75% |
| 27 | 27 | 29 | 93% | 77-99% |
| 28 | 67 | 186 | 36% | 29-43% |
| 29 | 72 | 186 | 39% | 32-46% |
| 30 | 22 | 186 | 12% | 8-17% |
| 31 | 17 | 186 | 9% | 5-14% |
| 32 | 28 | 186 | 15% | 10-21% |
| 33 | 14 | 186 | 8% | 4-12% |
| 34 | 18 | 186 | 10% | 6-15% |
| 35a | 91 | 186 | 49% | 42-56% |
| 35b | 31 | 186 | 17% | 12-23% |
| 35c | 77 | 186 | 41% | 34-49% |
| 36 | 75 | 186 | 40% | 33-48% |
| 37 | 44 | 186 | 24% | 18-30% |
| 38 | 85 | 186 | 46% | 38-53% |
| 39 | 69 | 186 | 37% | 30-44% |
| 40 | 13 | 186 | 7% | 4-12% |
| 41 | 2 | 186 | 1% | 0-4% |
| 42 | 18 | 186 | 10% | 6-15% |
